# Efficient nutrient management for enhancing crop productivity, quality and nutrient dynamics in lentil (*Lens culinaris* Medik.) in the semi-arid region of northern India

**DOI:** 10.1371/journal.pone.0280636

**Published:** 2023-02-10

**Authors:** Sandeep Kumar, Surender Kumar Sharma, Anil Kumar Dhaka, Sandeep Bedwal, Seema Sheoran, Ram Swaroop Meena, Chetan Kumar Jangir, Dinesh Kumar, Rakesh Kumar, Ram Dhan Jat, Ajit Kumar Meena, Ahmed Gaber, Akbar Hossain

**Affiliations:** 1 ICAR-Indian Agricultural Research Institute, Regional Station, Karnal, India; 2 Department of Agronomy, Chaudhary Charan Singh Haryana Agricultural University, Hisar, India; 3 Department of Soil Science, Chaudhary Charan Singh Haryana Agricultural University, Hisar, India; 4 Department of Agronomy, Institute of Agricultural Sciences, Banaras Hindu University, Varanasi, India; 5 ICAR-National Research Centre on Seed Spices, Tabiji, Ajmer, India; 6 ICAR-India Institute of Soil and Water Conservation, Research Centre, Datia, Madhya Pradesh, India; 7 Department of Microbiology, Chaudhary Charan Singh Haryana Agricultural University, Hisar, India; 8 Department of Soil Science and Agricultural Chemistry, Maharana Pratap University of Agriculture and Technology, Udaipur, Rajasthan, India; 9 Department of Biology, College of Science, Taif University, Taif, Saudi Arabia; 10 Division of Soil Science, Bangladesh Wheat and Maize Research Institute, Dinajpur, Bangladesh; Directorate of Rapeseed-Mustard Research, INDIA

## Abstract

Various faulty farming practices and low-performance cultivars selection are reducing crop yields, factor productivity, and soil fertility. Therefore, there is an urgent need to achieve better nutrient dynamics and sustainable production by selecting more nutrient-responsive cultivars using efficient nutrient management. The present experiment aimed to enhance crop productivity, seed quality, nutrient efficiency, and soil nutrient dynamics through efficient nutrient management under different lentil cultivars. The experiment was laid out in a split-plot design, assigning three cultivars (viz. Sapna, Garima, and HM-1) in the main plots and ten nutrient management practices in the sub-plots, replicating them thrice. Results revealed that cultivar HM-1 recorded significantly higher seed yield (1.59–1.61 Mg ha^-1^) and the uptake of N (67.2–67.6 kg ha^-1^), P (6.8–7.0 kg ha^-1^), K (13.8–13.9 kg ha^-1^), Zn (60.4–61.1 g ha^-1^), and Fe (162.5–165.2 g ha^-1^) in seed compared to Sapna and Garima. Also, the cultivar HM-1 was more efficient in terms of partial factor productivity for NPK (PFP; 24.27–24.59 kg kg^-1^), partial nutrient balance (PNB; 2.09–2.13 kg kg^-1^) and internal utilisation efficiency (IUE; 11.64–11.85 kg kg^-1^). The study showed that the lentil cultivar HM-1 could be successfully grown by substituting 50% RDN with organic manures, *i*.*e*., vermicompost, without compromising crop productivity and soil fertility, thereby sustaining soil-human-environment health.

## 1. Introduction

After the green revolution, the productivity of crops was significantly increased by using chemical fertilizers. Consumption of nitrogen (N) increased by more than nine times during this period. As of 2020, the agricultural consumption of N, phosphorus (P_2_O_5_), and potassium (K_2_O) has reached 113.3, 48.1, and 39.2 Mt, respectively [1, 2]. Due to this, global food grain production and productivity have increased by 3.4 and 2.25 times during the last six decades. However, due to long-term imbalance and overuse, the cultivated soil has become infertile and unproductive, and production costs have increased (30% of the entire cost of agricultural output). Chemical fertilizers have a damaging effect on soil, the environment, and human health, all under constant influence. Continuous use of fertilizers on the same piece of land increased soil compactness, polluted groundwater, and released greenhouse gases (GHGs), causing severe hazards to natural resources [3, 4]. Prioritizing chemical fertilizers over organic nutrition exhausted most of the soil’s organic matter, and its nutrient stock has been dramatically depleted [5, 6]. This reduces soil health, wastes valuable resources, and severely pollutes ecological sustainability when concern for food security and environmental safety is at its peak [7–14].

To meet these growing concerns about soil health, nutrient dynamics, and sustainability of food systems, applying organic manures to soil is far better than directly feeding chemical fertilizers to cultivars for better performance [15–19]. However, the use of organic manures in crop production has been in practice since ancient times. But, after the introduction of inorganic fertilizers during the 1960s, the bulky organic manures were neglected and considered a second alternative source to supply selected cultivars’ nutrients. So now, it is high time to promote biological agriculture by partially replacing chemical fertilizers with a combination of suitable varieties to protect soil-plant-human-environment health from further degradation. However, no single source of crop nutrients can meet the entire nutrient demands of the cultivars. Hence, integrating organic manures with synthetic fertilizers holds great promise in meeting crops’ nutrient demands as per the genotype selection [20, 21]. Therefore, it also helps to maintain crop productivity at higher levels with an overall improvement in the quality of the resource base and the farmer’s economics.

Grain legumes have been widely recognized for their vital role in addressing global food supply challenges, soil–environmental health, and nutritional security from the beginning [22]. These crops contribute about 10% of the daily protein and 5% of the energy intake and hence are particularly important for food security. For millennia, farmers have needed to practice the selected varieties with better crop nutrition to maintain and enhance soil health, restore degraded soils and improve overall human well-being [23]. Better responsive varieties have proven a boon to restoring soil’s inherent physio-chemical and biological properties to maintain its fertility and quality owing to their biological nitrogen fixation (BNF) ability [24]. Thus, legume crops help to reduce the heavy load of chemical fertilizers in the cereal-based cropping system, especially nitrogenous fertilizers. Several studies have been conducted to quantify the significance of grain legumes in increasing system productivity, soil quality, and manifest sustainable production [25, 26]. However, efficient nutrient management encompassing organic sources of nutrients in these crops has been ignored recently [27–36].

Overall, a better understanding and selection of the best stack cultivar with improved seed yield, more micronutrient accumulation, and enhanced NUE may prove beneficial to generating the highest benefits to the environment and society [37]. To explore this, the present experiment was designed to assess the impact of integrated nutrient management on crop yield, nutrient dynamics, and soil health using three different lentil cultivars. The study investigates the interaction of lentil cultivars with nutrient management levels for the production of plant biomass or yield, as well as the improvement of soil, food, and nutrient dynamics in the agroecosystem.

## 2. Materials and methods

### 2.1 Experimental site and climate condition

**Fig 1:**
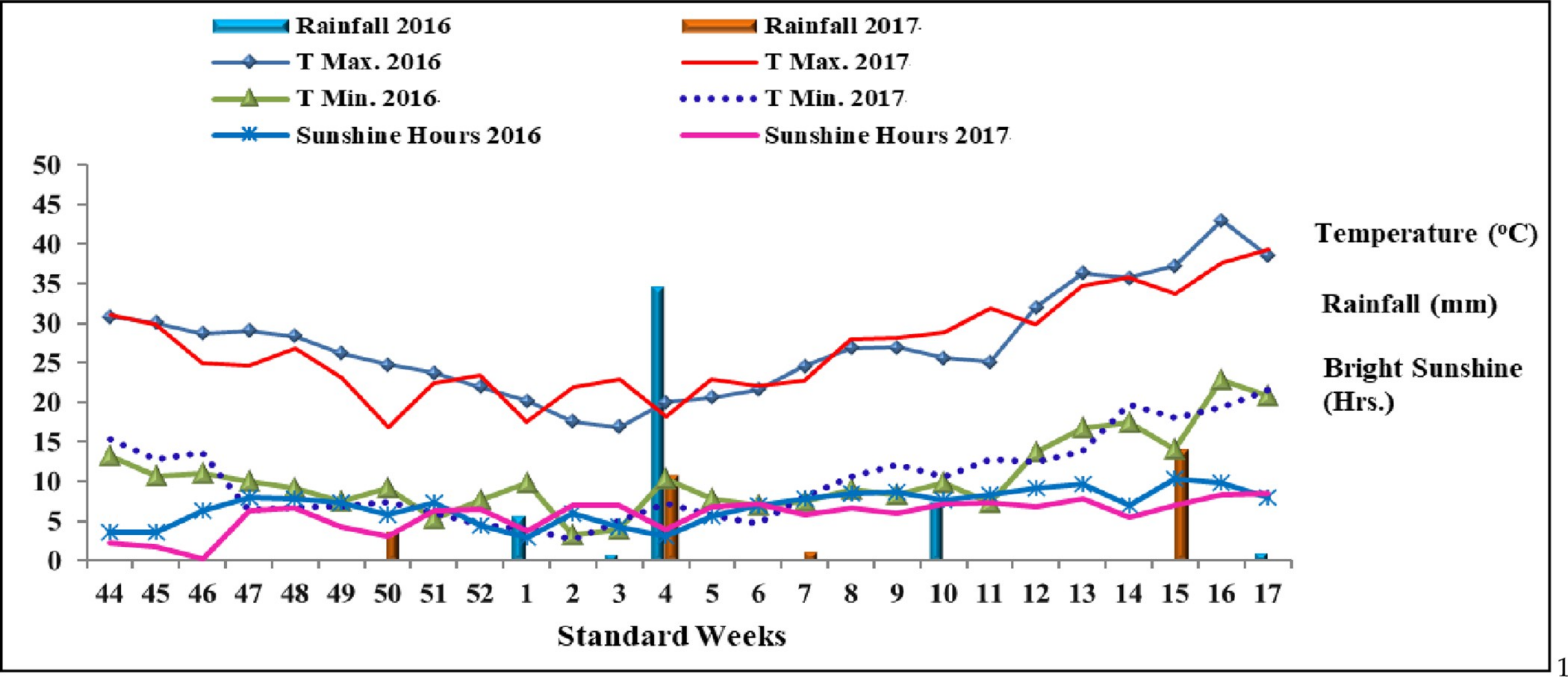
The average weather data of standard meteorological weeks of 2016 and 2017 during the crop duration.

The current field experiment was conducted at the Pulse Research Farm, Chaudhary Charan Singh Haryana Agricultural University (CCS HAU), Hisar, in the Haryana state of India, during two consecutive *Rabi* seasons of 2016 and 2017. The experimental site is stationed at 29°10’N latitude, 75°46’E longitude, with an elevation of 215.2 meters above mean sea level. The region’s climate is typically semi-arid tropical and sub-tropical, with hot and dry summers and frigid winters. The area receives approximately 485 mm of annual rainfall (mean of the last 30 years). Of these, about 78% occur between June and September, 3% between October and November (post-monsoon), 7% between December and February (winter season) and the remaining 12% between March and May (summer/pre-monsoon). Over the last 30 years, the average maximum temperature has been 31.5°C, while the average annual lowest temperature remained 16.5°C. Fig 1 shows the average weather data for standard meteorological weeks (during the crop duration) in 2016 and 2017.

### 2.2 Treatments and layout details

The treatments were laid out following the standard procedure of split-plot design by replicating three times. Three cultivars, *viz*., Sapna, Garima, and HM-1 (Haryana Massar 1) released from CCS HAU Hisar at different periods, *i*.*e*., Sapna in 1991, Garima in 1996, and HM-1 in 2006 were utilized in the current study. Sapna and Garima cultivars have erect plants with dark green foliage and bold flat shapes of seeds. While on the other hand, the cultivar HM-1 have light green foliage, smaller leaves and a more compact plant with less spread and smaller seeds. All three cultivars were assigned to main plots while ten nutrient management levels *viz*.; N1) control (no fertilization), N2) 100% recommended dose of fertilizers (RDF) (20:40 kg ha^-1^ –N:P_2_O_5_), N3) vermicompost (VC) at 2 t ha^-1^, N4) 50% RDN (recommended dose of nitrogen) + 100% RDP (recommended dose of phosphorus) + VC at 1 t ha^-1^, N5) RDF + 0.5% ZnSO_4_, N6) RDF + 0.5% FeSO_4_, N7) RDF + 0.5% ZnSO_4_ + 0.5% FeSO_4_, N8) 50% RDN + 100% RDP + VC at 1 t ha^-1^ + 0.5% ZnSO_4_, N9) 50% RDN + 100% RDP + VC at 1 t ha^-1^ + 0.5% FeSO_4_, and N10) 50% RDN + 100% RDP + VC at 1 t ha^-1^ + 0.5% ZnSO_4_ + 0.5% FeSO_4_ was allocated in sub-plots. ZnSO_4_ and FeSO_4_ foliar sprays were done twice during the pre-flowering and pod formation stages to observe their effect on Zn and Fe concentrations in developing lentil seeds. The entire amount of VC and chemical fertilizers were incorporated into the soil at the seeding time. The average concentration of N, P, K, Zn, and Fe in vermicompost was 1.15, 0.55, 1.08, 0.06, and 0.38 per cent, respectively.

### 2.3 Crop establishment and management

The experimental site was thoroughly ploughed using a tractor-drawn soil-turning mould board plough in the summer (June) to expose weed rhizomes and incorporate and destroy already-grown weeds and insect pests concealed in the soil. During the first week of November in both years, heavy pre-sowing irrigation was applied to enable effective ploughing and to ensure appropriate soil moisture for seed germination, establishment and subsequent plant growth. It was followed by two harrowing and a cross-ploughing with a cultivator, and the soil was well-levelled just before sowing. Furthermore, according to the layout plan, the field was divided into different experimental units and separated by 0.80 m wide and 0.20 m high earthen bunds. The crop was seeded using an indigenous plough at a row spacing of 22.5 cm and a depth of 5–6 cm on 29^th^ November and 02^nd^ December 2016 and 2017, respectively. A good amount of rainfall occurred during the reproductive stages of the crop during both years (Fig 1, standard week 4). Hence, no additional irrigation was given. Manual weeding was done twice, 4 and 7–8 weeks after sowing, with the help of *kasola* to eradicate and control existing weed flora in the field.

### 2.4 Soil sampling and analysis

An initial standard soil sample was collected from the top 15 cm of soil using a sampling auger before the initiation of the experiment. About 15 sub-samples (cores) were randomly collected in a zigzag pattern from various physiographic places across the experimental field and blended to create a representative (composite) sample delineating the entire experimental area. Each experimental plot was divided into four grids for soil sampling after the first-year crop harvest and before seeding the second-year crop. The soil was collected and composited appropriately within each grid cell. Each plot’s soil samples were combined entirely on a clean polythene sheet, and the mass was reduced by quadrating; consequently, only around 0.5 kg of the composite sample was kept and properly tagged and labelled. The samples were spread out on plastic paper for air drying in the shade and gently mashed with a wooden mortar pestle after air drying and sieved through a 2 mm sieve. Soil samples were analyzed in the laboratory for their chemical properties as per standard protocol, including the following formulas:

$$Organic\;carbon\;in\;soil\;(\%)=\frac{(Blank\;reading-sample\;reading)\times 0.003\times 100}{2\times weight\;of\;soil\;sample\;taken}\times 100$$ (Eq 1)

$$Zn/Fe\;content\;in\;soil\;(mg\;kg^{‐1})=(Sample\;reading–blank\;reading)\times dilution\;factor$$ (Eq 2)

The laboratory analysis showed that the soil of the experimental site was sandy loam in texture (International pipette method) [38] and saline in reaction (pH: 8.2±0.1, Glass electrode pH meter method) [39]. It was low in organic carbon (0.42%, Rapid titration method) [40] and available N (137.0±0.5 kg ha^-1^, Alkaline permanganate method) [41]; medium in available P_2_O_5_ (14.6±0.4 kg ha^-1^, Olsen’s method) [42], DTPA (diethylene triamine penta acetic acid)-extractable Zn (0.70±0.01 mg kg^-1^) and Fe (6.81±0.1 mg kg^-1^) [43], and high in exchangeable K_2_O (416.0±0.5 kg ha^-1^, Flame photometric method) [39]. Inductively coupled plasma mass spectrometry (ICP–MS) was used to determine Zn and Fe.

### 2.5 Plant sampling and analysis

Proper sampling is essential for obtaining reliable plant analysis results. At crop harvesting, seed and stover samples from each experimental plot were collected, representing the condition of all plants in that particular plot. The stover samples encompass the whole plant parts, *i*.*e*., stem, branches, leaves, etc. The collected samples were dried in an oven at 65°C for 24–72 hours to get a consistent weight. Mechanical grinding of seed and stover materials was carried out with stainless mills to pass a 60-mesh sieve, as most analytical procedures require grinding a dry sample. The grinded seed and stover materials were used for chemical analysis per the standard protocol, and the required calculations were done using the following formulas [14].


$$N\;uptake\;in\;seed/stover\;(kg\;ha^{‐1})=\%\;N\;in\;seed/stover\times seed/stover\;yield\;(kg\;ha^{‐1})$$ (Eq 3)

Proteincontentinseed(%)=%Ninseed×6.25 (Eq 4)

$$P\;uptake\;in\;seed/stover\;(kg\;ha^{‐1})=\%\;P\;in\;seed/stover\times seed/stover\;yield\;(kg\;ha^{‐1})$$ (Eq 5)

$$K\;uptake\;in\;seed/stover\;(kg\;ha^{‐1})=\%\;K\;in\;seed/stover\times seed/stover\;yield\;(kg\;ha^{‐1})$$ (Eq 6)

$$Zn/Fe\;content\;in\;seed/stover\;(mg\;kg^{‐1})=(Sample\;reading–blank\;reading)\times dilution\;factor$$ (Eq 7)

$$Zn/Fe\;uptake\;in\;seed/stover\;(g\;ha^{‐1})=Zn/Fe\;content\;in\;seed/stover\times seed/stover\;yield\;(kg\;ha^{‐1})$$ (Eq 8)

Partialfactorproductivity(PFP)(kgseed/kgnutrient)=Y/F (Eq 9)

$$Physiological\;efficiency\;(PE)=Y-Y_{0}/U-U_{0}$$ (Eq 10)

$$Agronomic\;efficiency\;(AE)(kg\;seed/kg\;nutrient)=Y–Y_{0}/F$$ (Eq 11)

Internalutilizationefficiency(IUE)=Y/U (Eq 12)

$$Partial\;nutrient\;balance\;(PNB)(kg\;nutrient/kg\;nutrient)=U_{H}/F$$ (Eq 13)

$$Apparent\;recovery\;efficiency\;(ARE)=U-U_{0}/F$$ (Eq 14)

Where, Y = Yield of the harvested portion of the crop with nutrient applied; Y_0_ = Yield with no nutrient applied; F = amount of nutrient applied; U = Total nutrient uptake in aboveground crop biomass with nutrient applied; U_0_ = Nutrient uptake in aboveground crop biomass with no nutrient applied; U_H_ = Nutrient content of the harvested portion of the crop.

### 2.6. Statistical analysis

The Statistical Analysis System (SAS) and ’R’ software were used to analyses variance (ANOVA) and statistical parameters [44, 45]. Two-way ANOVA was used to determine the statistical significance of the differences between the mean treatment values. Treatment means were compared at a 5% level of significance using the least significant difference (LSD) test to decode the significance of treatment effects.

## 3. Results

### 3.1 Crop yield

**Fig 2:**
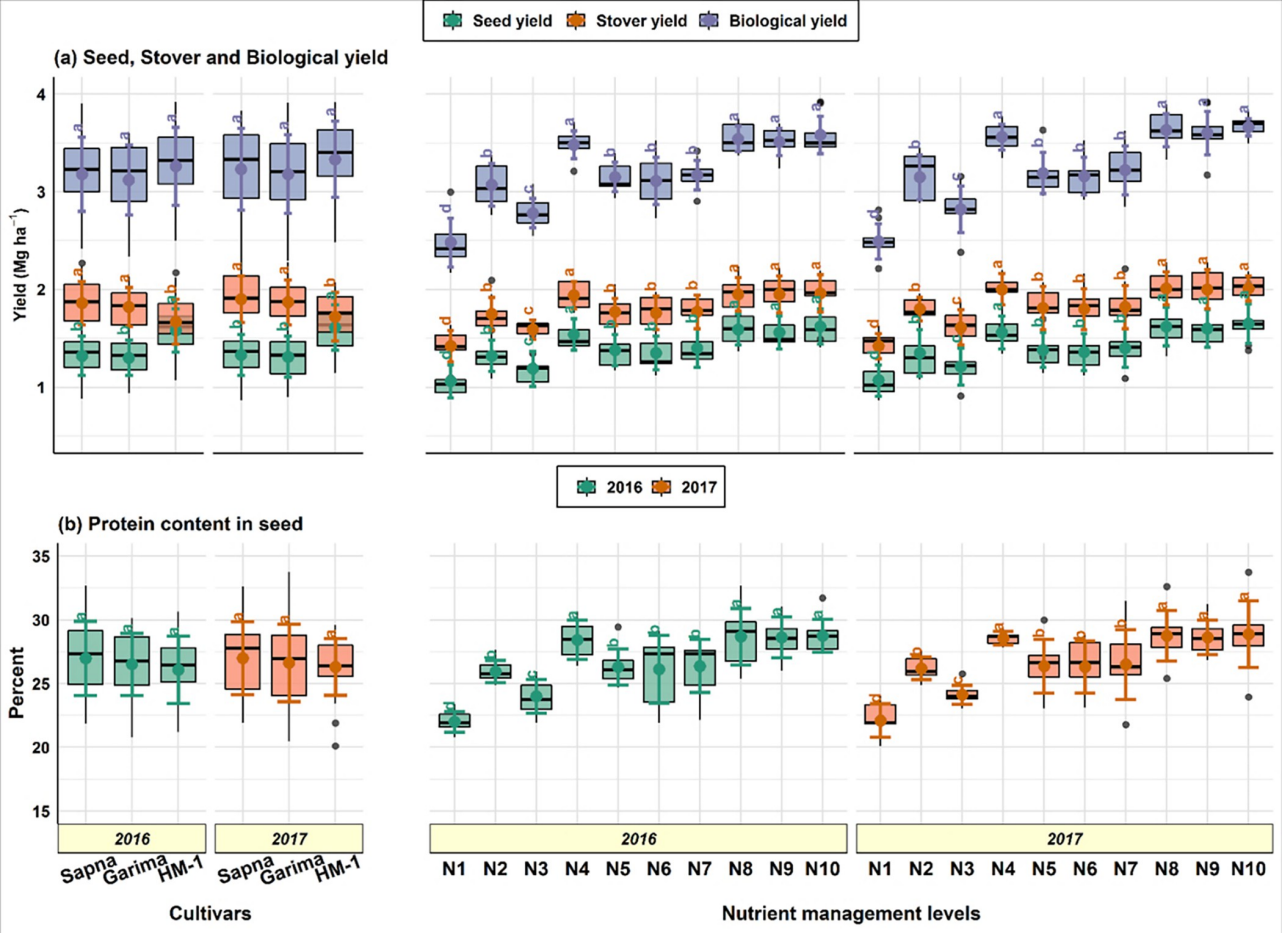
Crop yield (a) and seed protein content (b) of lentil cultivars under different nutrient management levels. *^a–d^ Different letters in the same column indicate a significant difference at the 0.05 probability level, *NS–non-significant. N1: control; N2: 100% RDF; N3: VC at 2 t ha^-1^; N4: 50% RDN + 100% RDP + VC at 1 t ha^-1^; N5: RDF + 0.5% ZnSO_4_; N6: RDF + 0.5% FeSO_4_; N7: RDF + 0.5% ZnSO_4_ + 0.5% FeSO_4_; N8: 50% RDN + 100% RDP + VC at 1 t ha^-1^ + 0.5% ZnSO_4_; N9: 50% RDN + 100% RDP + VC at 1 t ha^-1^ + 0.5% FeSO_4_; and N10: 50% RDN + 100% RDP + VC at 1 t ha^-1^ + 0.5% ZnSO_4_ + 0.5% FeSO_4_.

Cultivars have always been a significant contributor to crop production. In the present experiment, considerable variations were noted among lentil cultivars regarding seed, stover, and biological yield. Cultivar HM-1 recorded a significantly higher seed yield (1.59–1.61 Mg ha^-1^) compared to cultivar Sapna (1.31–1.33 Mg ha^-1^) and Garima (1.30 Mg ha^-1^). Although, the seed yield in the latter two cultivars was statistically similar (*p≤*0.05) (Fig 2A). In contrast, the cultivar Sapna (1.86–1.90 Mg ha^-1^) was statistically identical to Garima (1.82–1.87 Mg ha^-1^) produced significantly more stover than cultivar HM-1 (1.68–1.73 Mg ha^-1^). The biological yield of lentils remained statistically unaffected, but the cultivar HM-1 (3.27–3.34 Mg ha^-1^) had a numerically higher biological yield than the cultivars Sapna and Garima.

Among nutrient management practices, N10 produced the highest seed (1.63–1.65 Mg ha^-1^), stover (1.96–2.02 Mg ha^-1^), and biological (3.58–3.68 Mg ha^-1^) yields (Fig 2A). This was significantly superior to RDF with or without application of ZnSO_4_ and/or FeSO_4_ (N2, N5, N6, N7), vermicomposting at 2 t ha^-1^ (N3) and control one (N1). The plots with no nutrients (control) addition reported significantly lowest seed (1.06–1.07 Mg ha^-1^), stover (1.42–1.43 Mg ha^-1^), and biological (2.48–2.50 Mg ha^-1^) yields during both years. The extent of the increment in Yield in N10 was 53.3–60.4% for seed, 37.9–41.3% for stover, and 47.4–52.2% for biological Yield over their control.

### 3.2 Protein content

Across the cultivars, the protein concentration in lentil seeds ranged from 26.1 to 27.0% during the first year and 26.3 to 27.0% during the second year of study. The influence of cultivars on protein content in seed was observed to be non-significant (Fig 2B). Still, it followed a trend in protein content, with Sapna (27.0%) being on the higher side and HM-1 (26.1–26.3%) on the lower side. Various treatments of nutrient management exerted a significant influence on the protein content of the seed. The highest protein content (28.8–28.9%) was recorded in N10, which was statistically similar (*p≤*0.05) to N9 (28.6%), N8 (28.7–28.8%), and N4 (28.4–28.6%). Similarly, significantly higher protein content in seed was retained in treatments N10, N9, N8, and N4 compared to the remaining approaches. The control plot has the lowest protein content (22.0–22.1%) significantly compared to all the nutrient-applied plots. In N10, the increase in protein content was 30.4–30.5% over control and 10.3–10.7% over RDF (N2). Also, there was a positive correlation (0.71) between the amount of N added through different treatments and seed protein content on an average of both years.

### 3.3 Nutrient content

Throughout both years of the trial, the NPK content of lentil cultivars’ seeds and stover was nearly constant (Tables 1 and 2). Across the nutrient management treatments and crop growing years, the concentration of NPK in seed and stover significantly varied. It ranged from 3.52–4.62 (N), 0.37–0.45 (P), and 0.84 to 0.89% (K) in seed, and 0.87–1.24, 0.20–0.26, and 1.44–1.52% for N, P, and K, respectively, in stover.

**Table 1:** Nutrient content in the seed of lentil cultivars under different nutrient management levels.

Treatments	N (%)		P (%)		K (%)		Zn (mg kg^-1^)		Fe (mg kg^-1^)	
	2016	2017	2016	2017	2016	2017	2016	2017	2016	2017
**Cultivars**										
Sapna	4.32	4.32	0.43	0.44	0.87	0.89	38.24	38.31	103.27	104.63
Garima	4.24	4.26	0.42	0.43	0.86	0.87	37.12	38.18	102.73	103.98
HM-1	4.17	4.21	0.40	0.42	0.85	0.88	38.05	38.09	101.70	102.74
SEm±	0.06	0.07	0.01	0.01	0.02	0.01	0.34	0.36	1.70	2.74
LSD (*p≤*0.05)	*NS	NS	NS	NS	NS	NS	NS	NS	NS	NS
**Nutrient management practices**										
N1	3.52^d^	**Nutrient management practices**	0.37^b^	0.38^b^	0.84	0.86	36.94^b^	36.97^b^	99.11^b^	99.57^b^
N2	4.15^b^	3.52^d^	0.41^ab^	0.42^ab^	0.85	0.87	37.15^b^	37.03^b^	99.14^b^	99.61^b^
N3	3.84^c^	3.86^c^	0.40^ab^	0.41^ab^	0.88	0.89	37.35^b^	37.47^b^	99.36^b^	99.82^b^
N4	4.55^a^	4.57^a^	0.44^a^	0.44^a^	0.87	0.88	37.27^b^	37.23^b^	99.28^b^	99.73^b^
N5	4.21^b^	4.22^b^	0.41^ab^	0.42^ab^	0.85	0.87	39.54^a^	39.66^a^	99.16^b^	99.64^b^
N6	4.18^b^	4.21^b^	0.42^a^	0.42^ab^	0.84	0.86	37.18^b^	37.05^b^	108.91^a^	109.85^a^
N7	4.22^b^	4.24^b^	0.41^ab^	0.43^a^	0.85	0.87	39.57^a^	39.68^a^	108.95^a^	109.88^a^
N8	4.59^a^	4.60^a^	0.43^a^	0.44^a^	0.87	0.88	39.68^a^	39.79^a^	99.25^b^	99.75^b^
N9	4.58^a^	4.58^a^	0.43^a^	0.44^a^	0.86	0.89	37.29^b^	37.25^b^	109.18^a^	109.98^a^
N10	4.60^a^	4.62^a^	0.44^a^	0.45^a^	0.87	0.88	39.72^a^	39.82^a^	109.25^a^	110.01^a^
SEm±	0.11	0.12	0.01	0.02	0.03	0.02	0.66	0.74	3.27	3.44
LSD (*p≤*0.05)	0.29	0.31	0.05	0.05	NS	NS	1.88	2.10	9.27	9.75
Cultivar×Nutrient	NS	NS	NS	NS	NS	NS	NS	NS	NS	NS

^a–d^ Different letters in the same column indicate a significant difference at the 0.05 probability level

*NS–non-significant

**Table 2:** Nutrient content in stover of lentil cultivars under different nutrient management levels.

Treatments	N (%)		P (%)		K (%)		Zn (mg kg^-1^)		Fe (mg kg^-1^)	
	2016	2017	2016	2017	2016	2017	2016	2017	2016	2017
**Cultivars**										
Sapna	1.13	1.16	0.24	0.25	1.50	1.53	25.19	26.12	215.7	219.2
Garima	1.12	1.14	0.24	0.25	1.47	1.50	25.11	26.02	214.6	217.8
HM-1	1.09	1.12	0.23	0.24	1.45	1.48	25.06	25.96	212.4	215.2
SEm±	0.02	0.03	0.003	0.004	0.02	0.03	0.23	0.25	3.5	3.7
LSD(0.05)	*NS	NS	NS	NS	NS	NS	NS	NS	NS	NS
**Nutrient management practices**										
N1	0.86^d^	0.88^d^	0.20^b^	0.21^b^	1.44	1.48	24.44^b^	25.20^b^	207.0^b^	208.6^b^
N2	1.09^b^	1.09^c^	0.23^ab^	0.24^b^	1.45	1.49	24.47^b^	25.24^b^	207.1^b^	208.7^b^
N3	0.98^c^	0.99^b^	0.22^b^	0.23^b^	1.51	1.53	24.60^b^	25.54^b^	207.5^b^	209.1^b^
N4	1.21^a^	1.22^a^	0.25^a^	0.25^a^	1.49	1.51	24.55^b^	25.38^b^	207.2^b^	208.9^b^
N5	1.10^b^	1.10^b^	0.23^ab^	0.25^a^	1.46	1.50	25.98^a^	27.03^a^	207.1^b^	208.8^b^
N6	1.10^b^	1.09^b^	0.23^ab^	0.24^ab^	1.45	1.50	24.49^b^	25.25^b^	227.5^a^	230.2^a^
N7	1.11^b^	1.11^b^	0.24^a^	0.24^ab^	1.46	1.49	26.00^a^	27.05^a^	227.6^a^	230.2^a^
N8	1.21^a^	1.23^a^	0.24^a^	0.25^a^	1.49	1.51	26.03^a^	27.12^a^	207.3^b^	209.0^b^
N9	1.22^a^	1.23^a^	0.24^a^	0.25^a^	1.48	1.52	24.56^b^	25.39^b^	228.0^a^	230.4^a^
N10	1.23^a^	1.24^a^	0.25^a^	0.26^a^	1.48	1.52	26.04^a^	27.14^a^	228.2^a^	230.5^a^
SEm±	0.03	0.03	0.01	0.01	0.04	0.05	0.44	0.50	5.8	6.1
LSD (0.05)	0.09	0.10	0.03	0.03	NS	NS	1.24	1.43	13.2	13.9
Cultivar × Nutrient	NS	NS	NS	NS	NS	NS	NS	NS	NS	NS

^a–d^ Different letters in the same column indicate significant difference at the 0.05 probability level

*NS–non-significant

The N concentration in seed and stover was statistically higher with the application of 50% RDN + 100% RDP + VC at 1 t ha^-1^ with or without ZnSO_4_ or FeSO_4_ (N10, N9, N8, N4) compared to control (N1), RDF (N2), and VC at 2 t ha^-1^ (N3). In seed, these treatments (N10, N9, N8, and N4) accumulated 29.1–30.7%, 9.10–10.8%, and 18.4–19.8% more N than N1, N2, and N3, respectively. While in the case of stover, the average increase in N content over N1, N2, and N3 during the growing years was 25.8–41.4, 11.0–13.8, and 13.0–25.5%, respectively. Similarly, significantly higher N content in seed and stover was observed in N2 compared to N3, which was statistically superior to the control. In the case of P, the N10 observed a significantly higher concentration in seed (0.44–0.45%) and stover (0.25–0.26%) only to control, while it remained statistically equal in all other treatments. The K concentration in seed and stover remained constant over the different levels of nutrients during both years. Furthermore, lentil cultivars did not respond to a twice-foliar spray of 0.5% ZnSO_4_ and FeSO_4_ individually or collectively in terms of improved N, P, and K content in seed and stover compared to non-sprayed treatments. The interaction effect of cultivars and nutrient management approaches was found to be non-significant concerning macro and micronutrient concentrations in the seed.

Like major nutrients, nutrient management practices significantly influenced the concentration of micronutrients (Zn and Fe) in seed and stover (Tables 1 and 2). The foliar application of ZnSO_4_ and/or FeSO_4_ significantly increased Zn and Fe concentrations in seed and stover over plots in which foliar spray was not done. Zn concentrations in seeds in N10 (39.72–39.82 mg kg^-1^), N8 (39.68–39.79 mg kg^-1^), N7 (39.57–39.68 mg kg^-1^) and N5 (39.54–39.66 mg kg^-1^) were statistically equal, but all these had significantly higher Zn content than the remaining treatments (36.94–37.47 mg kg^-1^).

The same trends were found for Zn content in lentil stover. The foliar Zn fertilization improved its concentration by 7.0–7.7% in seed and 6.3–7.7% in stover. Similarly, in the foliar spray of FeSO_4_, the Fe concentration in seed and stover was significantly increased. In this respect, N10 (109.25–110.01 mg kg-^1^), N9 (109.18–109.98 mg kg^-1^), N7 (108.95–109.88 mg kg^-1^), and N6 (108.91–109.85 mg kg^-1^) had significantly higher Fe concentrations in seed than the other treatments (99.11–99.82 mg kg^-1^). A similar pattern of Fe concentration was noted in lentil stover during both years. The foliar spray of FeSO_4_ (0.5%) at pre-flowering and podding stages enhanced Fe buildup in the seed and stover of lentil by 9.9–10.5% on average. The interaction effect of cultivars and nutrient management strategies was statistically non-significant for nutrient concentrations in lentil stover.

### 3.4 Nutrient uptake

**Fig 3:**
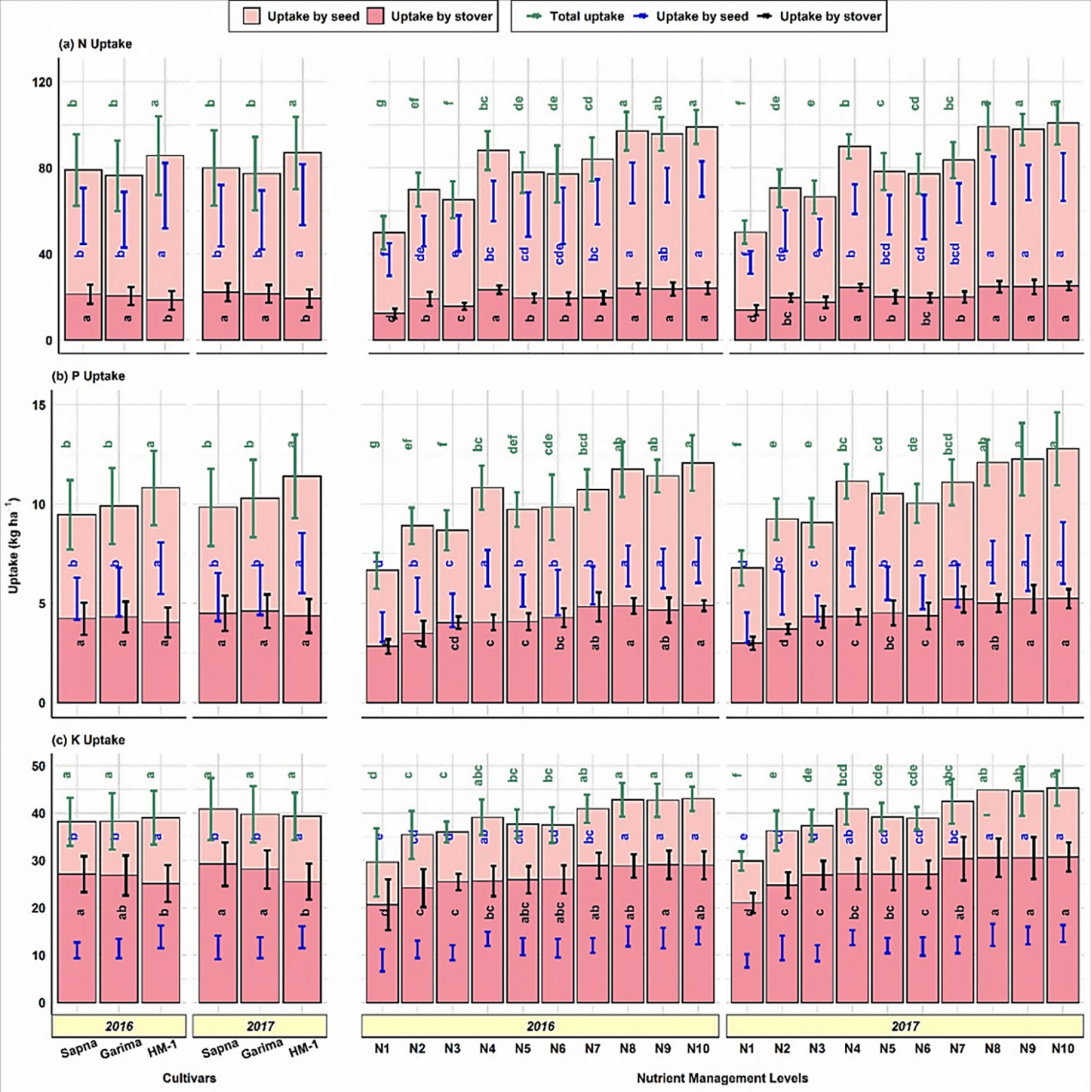
Nutrient uptake by seed and stover of lentil as influenced by cultivar selection and nutrient management levels.

Nutrient uptake in seed was significantly different among lentil cultivars (Fig 3). Cultivar HM-1 had a statistically more significant uptake of macro (NPK) as well as micronutrients (Zn, Fe) in seed than Sapna and Garima. The value of nutrient uptake by seeds with cultivar HM-1 was 67.2–67.6 kg ha^-1^ for N, 6.8–7.0 kg ha^-1^ for P, 13.8–13.9 kg ha^-1^ for K, 60.4–61.1 g ha^-1^ for Zn, and 162.5–165.2 g ha^-1^ for Fe. In contrast, the highest uptake of N (21.32, 22.19 kg ha^-1^), K (27.10, 29.21 kg ha^-1^), Zn (46.9, 49.7 g ha^-1^), and Fe (402.7, 418.2 kg ha^-1^) in stover was found in cultivar Sapna, followed by Garima in the respective years of study. A non-significant difference was observed among cultivars regarding P uptake in stover. Although cultivar Garima had numerically more P uptake, it was followed by Sapna and HM-1.

The NPK uptake by seed varied greatly with nutrient management levels, ranging from 37.4–74.9 and 36.2–75.7 for N, 3.82–7.17 and 3.79–7.55 for P, and 8.96–14.06 and 8.81–14.56 kg ha^-1^ for K (Fig 3). The maximum uptake of NPK by seed and stover was noticed in N10 compared to the remaining treatments except for N9, N8, and N4.

The uptake by seeds in N10 was 100.3–109.1, 87.7–99.2, and 56.9–65.3% higher for N, P, and K, respectively, compared to the control treatment. Similar to macronutrients, the uptake of Zn and Fe by seed and stover was also significantly higher in N10. However, the uptake of Zn by seed in N10 (64.6–65.5 g ha^-1^) was statistically similar to N8 (63.4–64.4 g ha^-1^) during both years. Also, the Zn uptake by stover in N10 (51.1–54.8 g ha^-1^) was statistically comparable with N8 (50.8–54.4 g ha^-1^), N9 (47.8–51.1 g ha^-1^), and N4 (47.6–50.9 g ha^-1^). In the case of Fe uptake by seed, N10 (177.9, 181.2 g ha^-1^) was statistically comparable with N9 (171.1 g ha^-1^) during the first year and with N9 (175.2 g ha^-1^) and N8 (161.8 g ha^-1^) during the second year of the experiment. Even in the case of Fe uptake by stover, the N10 (446.3–466.8 g ha^-1^) was statistically comparable with N9 (443.6–466.0 g ha^-1^), N8 (403.6–420.0 g ha^-1^), N7 (403.7–419.6 g ha^-1^), and N6 (401.8–418.9 g ha^-1^). Seeds’ Zn and Fe uptake were 65.6 and 72.8% (Zn) and 70.2 and 78.0% (Fe) higher in N10 than in control during both years, respectively. Stover uptake of Zn and Fe was 47.7–52.2% (Zn) and 51.9–56.5% (Fe) higher in N10 than in the control plots.

### 3.5 Total nutrient uptake

**Fig 4:**
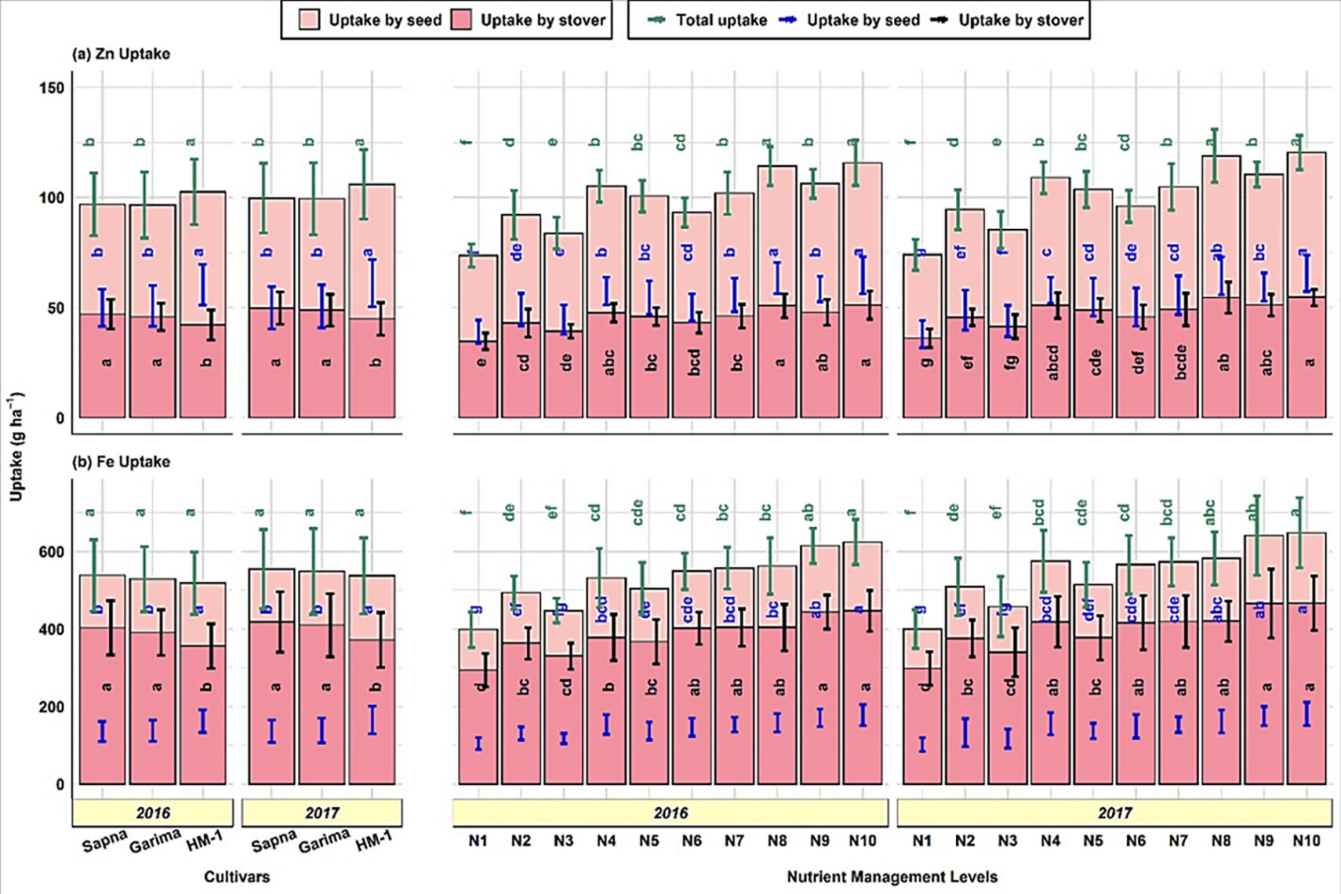
Total nutrient uptake (seed + stover) of lentil as influenced by cultivar selection and nutrient management levels.

Total uptake of N and P was significantly higher in cultivar HM-1 compared to Sapna and Garima during both years of investigation (Fig 4).

The cultivar HM-1 recorded 85.70–86.98 kg ha^-1^ N and 10.82–11.39 kg ha^-1^ P. The difference in terms of the total uptake of K, Zn and Fe of lentil cultivars was found to be non-significant during both years. The nutrient management practices significantly influenced the total uptake of all the nutrients (Fig 4). In this regard, the uptake of N (99.0, 100.8 kg ha^-1^), P (12.07, 12.79 kg ha^-1^), K (43.05, 45.26 kg ha^-1^), Zn (90.8, 120.3 g ha^-1^), and Fe (624, 647 g ha^-1^) was maximum in N10. However, the NP uptake in N10 was not significantly different from N8 and N9. Similarly, there was no significant difference in K uptake in N10, N8, N9, N7, and N4 during both years. The total uptake of Zn in treatment N10 was non-significant, with N8, N9, N7, N5, and N4 during the first year of the experiment and with N8 during the second year of the investigation. The total Fe uptake by N10 failed to reach a significant level with N9 during the first year and with N9, N8, and N4 during the second year of the study. A significantly lowest uptake of all nutrients was observed in the control (N1) plot during both years of study. The extent of the increase in total uptake of N, P, K, Zn and Fe in N10 concerning the absolute control was 98.64–101.04, 81.50–88.64, 45.44–51.57, 26.88–62.74, and 56.72–61.96%, respectively.

### 3.6 Soil chemical properties

**Fig 5:**
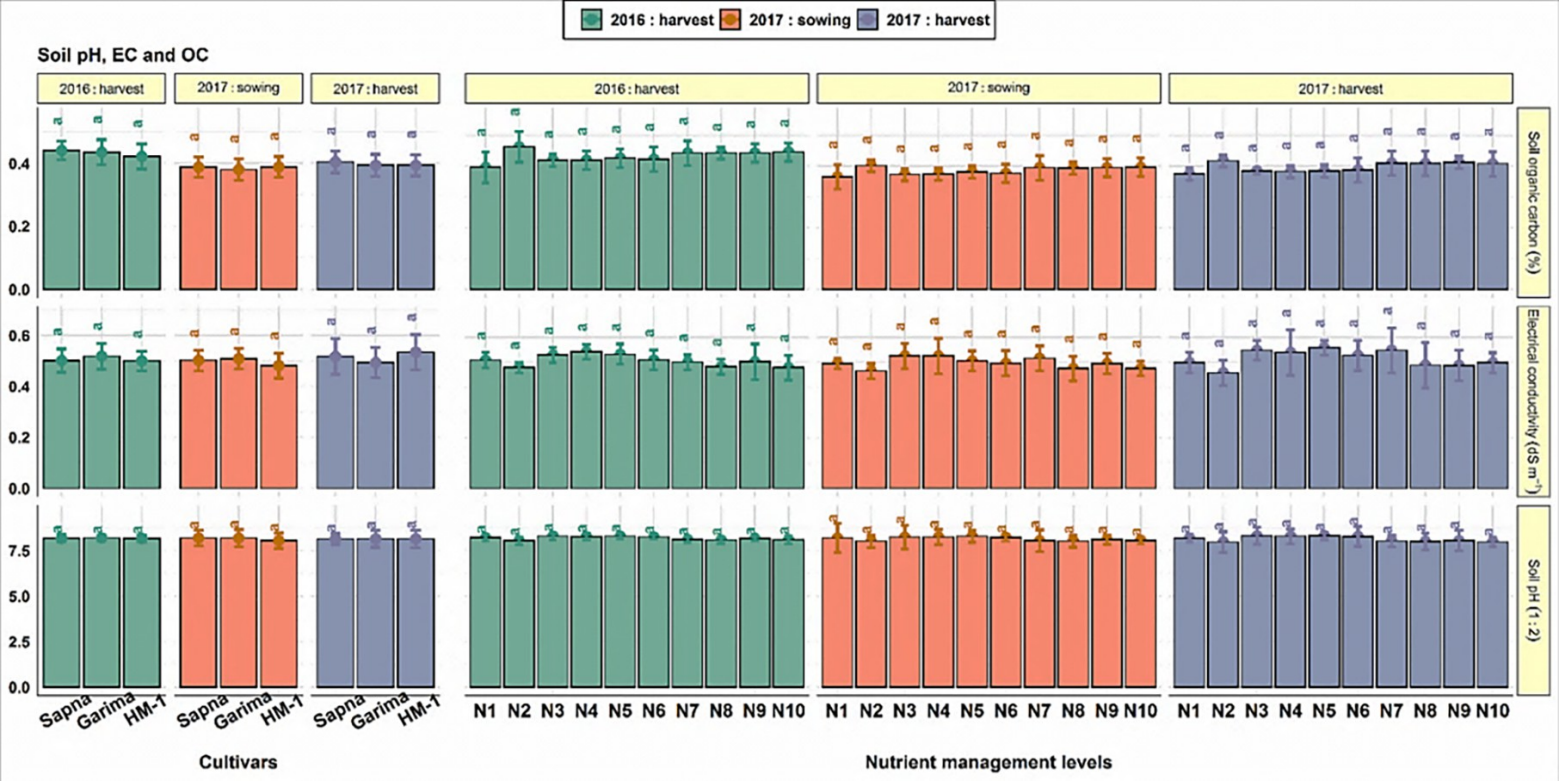
Soil pH, EC and organic carbon as influenced by cultivar selection and nutrient management levels.

The results (Fig 5) indicated that after crop harvest, the soil pH (1:2), EC, and OC remained almost unchanged from their corresponding initial values. Among cultivars, the values of all these soil parameters remained identical after crop harvest during both years concerning their initial values (at sowing).

**Fig 6:**
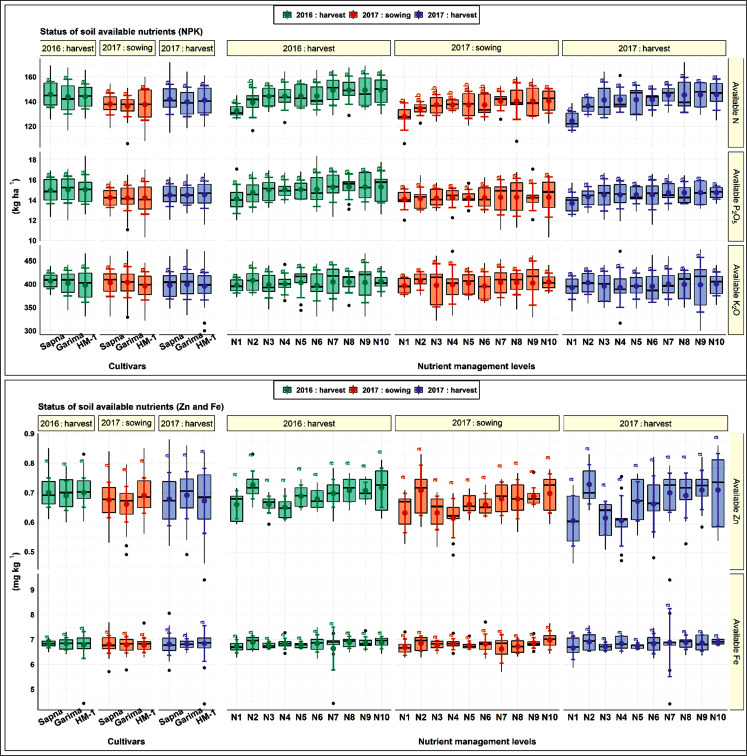
Status of soil available nutrients as influenced by cultivar selection and nutrient management levels.

Similarly, the nutrient management practices also failed to alter these soil chemical parameters to a significant level during both years. Although numerically, the soil pH and EC were slightly lower than their initial values in plots supplied with VC, chemical fertilizers slightly increased their values over control plots. Likewise, the VC slightly improved the soil C stock, and its extent varied with the amount of VC being used. The effect of nutrient management levels on all these parameters was non-significant. The observed data (Fig 6) showed that none of the lentil cultivars significantly influenced available NPK and DTPA-extractable Zn and Fe in soil.

The available soil N was improved considerably in N10 from N1 and N3 during both years. Compared to the initial value of available N (137 kg ha^-1^), *i*.*e*., before the start of the experiment, the N10 improved (149.3 kg ha^-1^) its status after harvesting crops in the first year. Afterwards, N concentration decreased and was found to be 140.5 kg ha^-1^ just before the start of the second-year experiment, which again increased to 145.5 kg ha^-1^ at crop harvest. However, after crop harvest, available N under RDF (N2) remained nearly the same as the initial value. In control plots, N content decreased over the initial value during both years. On the other hand, none of the nutrient management practices significantly affected available P and K as well as DTPA-extractable Zn and Fe in the soil at the end of the study. Also, during the fallow period between the harvesting of the first-year crop and the sowing of the second-year crop, the variations in available Zn and Fe in the soil were negligible.

### 3.7 Correlation analysis of soil properties

**Fig 7:**
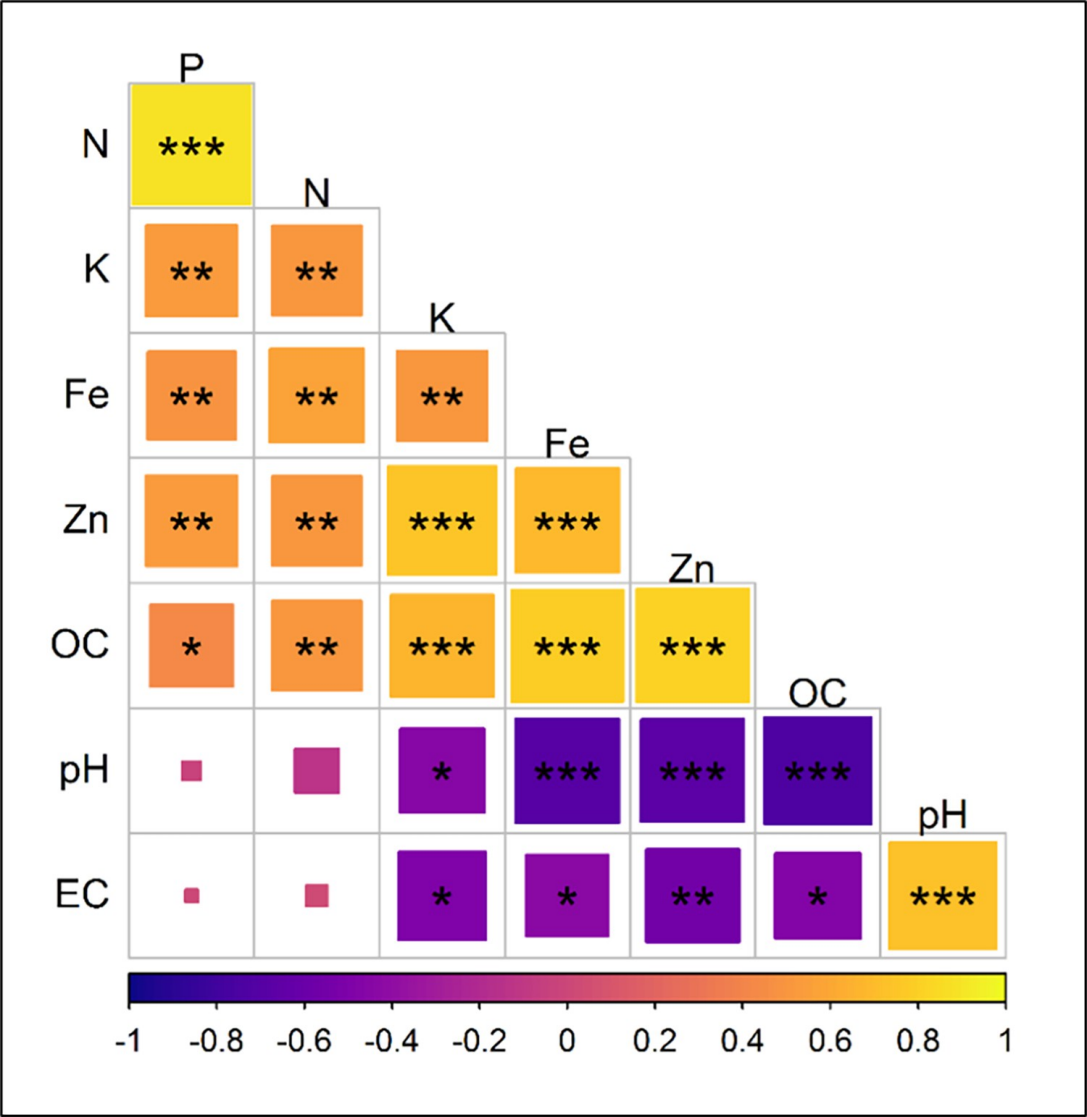
Correlation matrix of different soil properties based on R package.

The correlation matrix (Fig 7) highlights the most correlated variables among different soil parameters. A strong positive correlation was found in soil pH × EC, N × P, OC × Zn, OC × Fe, OC × K, Zn × K, and Zn × Fe, whereas a moderate positive correlation (0.50–0.65) exists in N × K, N × Fe, N × Zn, N × OC, P × K, P × Fe, P × Zn, and K × Fe.

Moreover, a strong negative correlation was found between soil pH × OC, pH × Zn, and pH × Fe, while EC × Zn has a moderate negative correlation. A weak negative correlation was found between pH × K, EC × K, EC × Fe, and EC × OC. Also, it was observed that pH × N, pH × P, EC × P, and EC × N have no significant correlation.

### 3.8 Principal component analysis for soil properties

**Fig 8:**
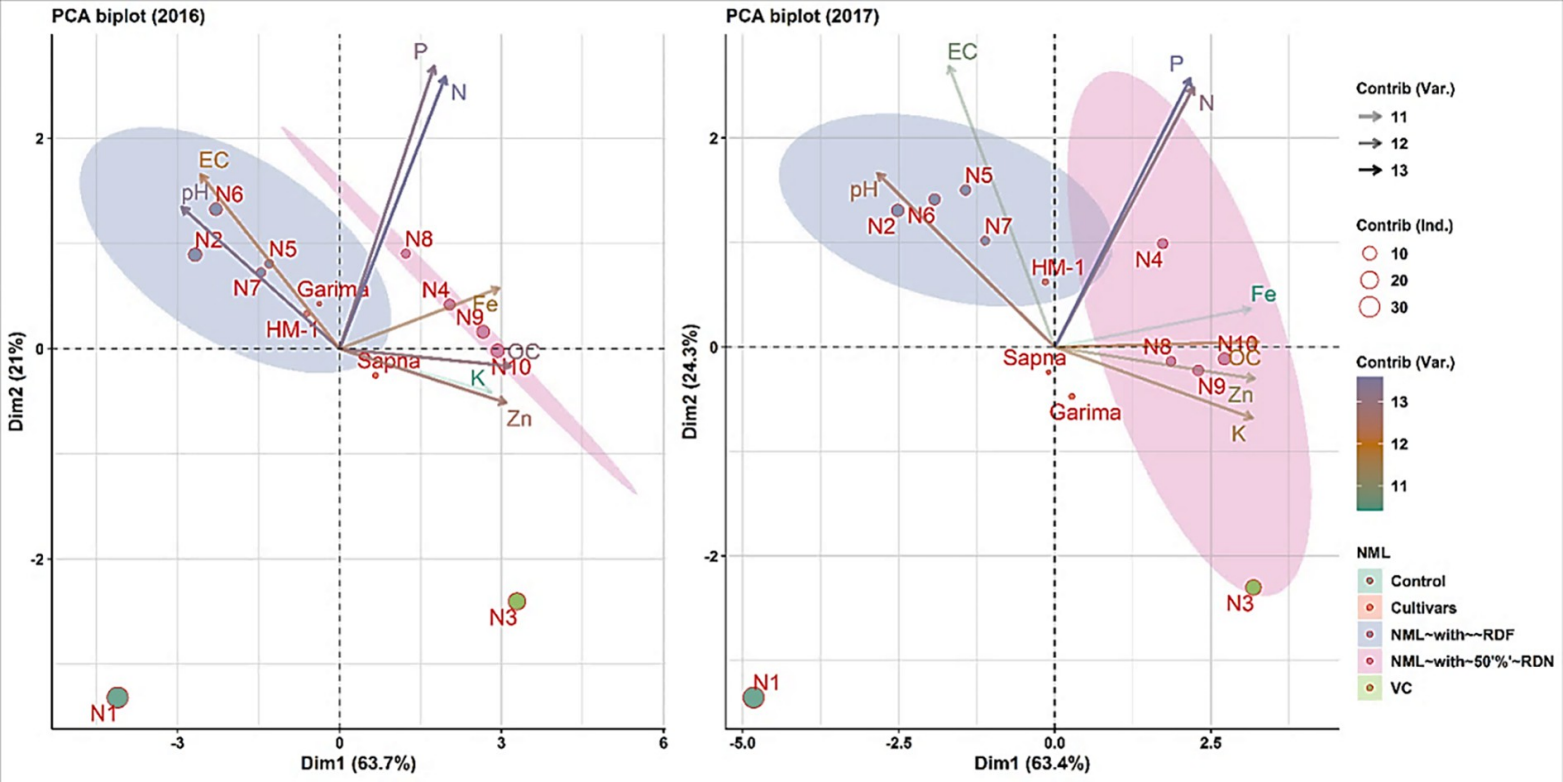
Graphic biplot for principal components (PC1 and PC2) for soil properties after harvest of first (A) and (B) second years.

The PCA (principal component analysis) was performed to identify any possible grouping among the diverse soil parameters observed against the different cultivars and nutrient management level loadings for variables and individuals for both years (Fig 8).

During 2016 and 2017, PC1 contributed 63.7, and 63.4% of the variability, while PC2 contributed 21, and 24.3% of the variability, respectively. Treatments N4, N8, N9, and N10 (treatments with 50% RDN) stayed in the right section of the PCA biplots. Nutrient management treatments N2, N5, N6 and N7 (treatments with 100% RDF with other combinations) were the individuals with negative loadings on PC1 and positive loadings on PC2. They stayed in the upper left section of the PCA biplots. Treatment N1 and N3 (no chemical fertilizers) stayed in different lower sections of the PCA biplot during both years. Varieties’ positions in the biplot indicate no influence of varieties on soil properties. Concerning the variables, it was noticed that pH and EC have negative loadings on PC1 and positive loadings on PC2 (upper left section of PCA biplots). In contrast, variables K and Zn showed loadings contradictory to pH and EC (lower right section of PCA biplots). Variables N, P, and Fe have positive loadings on both PCs (upper right section of PCA biplots). Furthermore, the shrinkage in the point size of individuals or the lessening length/colour intensity of the arrows of the variables signifies reduced variability, which is the instance reported with the individuals Sapna, Garima, HM-1, N5, and variables K, Fe, and Zn indicating that these variables/individuals have not contributed much to variability.

### 3.9 Nutrient use efficiencies

In the present experiment, NUEs were measured in terms of partial factor productivity (PFP), partial nutrient balance (PNB), internal utilization efficiency (IUE), physiological efficiency (PE), apparent recovery efficiency (ARE), and agronomic efficiency (AE). These parameters significantly (*p≤*0.05) differed across the lentil cultivars (Table 3).

**Table 3:** Effect of cultivars and nutrient management practices on nutrient use efficiencies (kg kg^-1^) for cumulative NPK.

Treatments	PFP		PNB		IUE		PE		ARE		AE	
	2016	2017	2016	2017	2016	2017	2016	2017	2016	2017	2016	2017
**Cultivars**												
Sapna	20.40^b^	20.33^b^	1.96^b^	1.99^b^	10.48^b^	10.46^b^	8.76^a^	10.80^a^	68	74	5.73	6.06
Garima	19.95^b^	19.98^b^	1.93^b^	1.97^b^	10.43^b^	10.46^b^	8.30^a^^b^	10.58^a^^b^	69	72	5.38	6.16
HM-1	24.27^a^	24.59^a^	2.09^a^	2.13^a^	11.64^a^	11.85^a^	8.08^b^	9.88^b^	70	73	5.53	6.14
SEm±	0.30	0.47	0.028	0.038	0.17	0.19	0.15	0.20	10	5	0.12	0.16
LSD (0.05)	1.19	1.89	0.11	0.13	0.69	0.72	0.55	0.62	*NS	NS	NS	NS
**Nutrient management**												
N1	-	-	-	-	-	-	-	-	-	-	-	-
N2	22.00^a^^b^^c^^d^	22.15^a^^b^^c^^d^	1.90^b^^c^	1.94^c^^d^	11.59^a^	11.64^a^	9.33^a^	12.26^b^^c^	47^d^^e^	49^e^^f^	4.33^d^	5.05^c^
N3	21.42^b^^c^^d^^e^	21.08^b^^c^^d^^e^	1.98^b^	2.03^b^^c^	10.90^a^^b^	10.72^a^^b^	5.74^e^	5.80^f^	43^e^	47^f^	2.36^e^	2.63^e^
N4	19.83^e^	20.04^e^	1.77^c^	1.83^d^	11.23^a^^b^	11.40^a^^b^	9.59^a^^b^	14.52^a^	67^b^	71^b^^c^^d^	6.21^a^^b^^c^	6.85^a^^b^^c^
N5	23.02^a^^b^	22.99^a^^b^	2.09^a^^b^	2.13^a^^b^	11.00^a^^b^	11.05^a^^b^	8.71^b^^c^	11.37^c^	65^c^^d^	69^c^^d^	5.35^b^^c^	5.88^b^^c^
N6	22.52^a^^b^^c^	22.57^a^^b^^c^	2.07^a^^b^	2.10^b^^c^	10.92^a^^b^	11.02^a^^b^	9.38^a^^b^	12.73^b^	64^c^^d^	66^d^^e^	4.85^c^^d^	5.47^c^
N7	23.47^a^	23.35^a^	2.26^a^	2.29^a^	10.40^b^	10.36^b^	7.20^d^	8.39^e^	83^a^^b^	84^a^^c^	5.80^a^^b^^c^^d^	6.25^a^
N8	20.55^d^^e^	20.83^c^^d^^e^	1.95^b^^c^	2.00^b^^c^	10.58^a^^b^	10.74^a^^b^	8.77^b^^c^	10.12^d^	84^a^^b^	89^a^	6.93^a^^b^	7.65^a^^b^
N9	20.13^d^^e^	20.51^d^^e^	1.93^b^^c^	1.99^b^^c^^d^	10.47^b^	10.69^a^^b^	8.13^c^	9.29^d^^e^	82^a^^b^^c^	87^a^^b^	6.50^a^^b^^c^	7.33^a^^b^^c^
N10	20.89^c^^d^^e^	21.16^b^^c^^d^^e^	1.98^b^	2.04^b^^c^	10.54^a^^b^	10.70^a^^b^	8.58^c^	9.31^d^^e^	87^a^	93^a^	7.26^a^	7.97^a^
SEm±	0.66	0.69	0.06	0.05	0.39	0.56	0.29	0.38	6.4	5.5	0.60	0.68
LSD (0.05)	1.89	1.97	0.19	0.16	1.07	1.21	0.73	0.92	18	16	1.72	1.94
Cultivar × Nutrient	NS	NS	NS	NS	NS	NS	NS	NS	NS	NS	NS	NS

^a–f^ Different letters in the same column indicate significant difference at the 0.05 probability level

*NS–non-significant

The cultivar HM-1 has the most significant impact on PFP (24.27–24.59 kg kg^-1^), PNB (2.09–2.13 kg kg^-1^) and IUE (11.64–11.85 kg kg^-1^) of lentils compared to cultivars Sapna and Garima, while the last two have more or less the same impact on all these parameters. At the same time, cultivar Sapna (8.76–10.80 kg kg^-1^) was found to be significantly more efficient in PE for NPK, although it was statistically similar to Garima. The NUEs in ARE and AE were not considerably affected due to cultivar selection. However, the superiority of cultivars in terms of ARE and AE differed in experimental years.

Using organic and inorganic fertilizers enhanced nutrient availability, SOM, microbial and enzymatic processes, and various soil physical qualities. This, in turn, increased biomass output, nutrient uptake, and, subsequently, use efficiency [46]. In this regard, the impact of N7 on the improvement of PFP (23.47 and 23.35 kg kg^-1^) and PNB (2.26 and 2.29 kg kg^-1^) of lentils was significantly highest. However, its impact was statistically comparable with N2, N5, and N6 in the case of PFP only. In contrast, N4 retained the lowest PFP (19.83 and 20.04 kg kg-1) and PNB (1.77 and 1.83 kg kg-1) significantly during both years, respectively. Furthermore, the significantly highest IUE for NPK was recorded in N2 (11.59 and 11.64 kg kg^-1^), while N7 has the lowest IUE (10.40 and 10.36 kg kg^-1^). In the case of AE and ARE, N10 was a significantly superior treatment, while N3 had the lowest values. The improvement in PE for NPK in treatment N4 was found to be the highest (9.59–14.52 kg kg^-1^), while N3 had the lowest value (5.74–5.80 kg kg^-1^).

The correlation between the amounts of nutrients supplied through different nutrient management practices (NPs) and nutrient use efficiencies was carried out. In this study, a strong positive correlation of NPs was found with AE (0.83, 0.84) and ARE (0.71), while it was partially positive with PE (0.29). Furthermore, there was a negative correlation between NPs and PFP (–0.78)/ PNB (–0.53), while partially negative between NPs and IUE (–20) irrespective of the production year. This means that the values of PFP, PNB, and IUE declined with an increase in the rate of cumulative NPK.

## 4. Discussion

### 4.1 Cultivars

#### 4.1.1 Crop yield

The lentil cultivar HM-1 produced a significantly higher seed yield (1.59–1.61 Mg ha^-1^) than Sapna (1.31–1.33 Mg ha^-1^) and Garima (1.30 Mg ha^-1^) (Fig 2A). Effective mobilization of dry matter production towards the sink (seed yield) is critical for economic yield. The superiority of cultivar HM-1 may be due to its more remarkable ability to convert assimilates into economic yield, which is linked to its differential gene expression and response to applied inputs. Furthermore, the higher Yield in HM-1 was also associated with its higher branch production and pods per plant, which contributed to the seed yield and its supremacy over Sapna and Garima. Various researchers have noted differences in seed output across different crops [47, 48] as well as lentil cultivars also [49, 50]. Sapna, shortly followed by Garima, produced substantially more stover than HM-1. This could be attributed to the plants’ rapid development and, as a result, greater biomass production of leaves. Sapna and Garima are two robust, bold-seeded cultivars that may have used their additional photosynthetic energy to boost their vegetative growth. While in cultivar HM-1, photosynthetic assimilates may have been translocated more in the sink region of the plant (*i*.*e*., seed) than in vegetative growth. Similar findings were reported by Biswas et al. [50]. In both years, the biological yield of lentils remained statistically equivalent across all cultivars. This was because cultivar HM-1 produced higher seed yields, and cultivars Sapna and Garima produced higher stover yields, as in Fig 2A.

#### 4.1.2 Nutrient content, uptake and seed protein

The concentration of NPKZn and Fe in seed and stover remained equal across the lentil cultivars (Tables 1 and 2). Haq et al. [51] and Köse et al. [52] found no significant trends in Zn and Fe concentrations in lentil seed and stover. Similarly, the influence of cultivars on seed protein content was observed to be non-significant. Nitrogen is the primary constituent of protein and directly regulates the protein concentration in the seed. Hence, protein content remained non-significant as seed N content was statistically comparable among cultivars, as shown in Table 1. Mondal et al. [53] also found statistically identical protein content in the seeds of different lentil cultivars. The results show that lentil cultivar HM-1 has a statistically greater uptake of macro (N, P, and K) as well as micronutrients (Zn and Fe) in seed than cultivars Sapna and Garima (Fig 3). The biomass yield and the nutrient concentration in the biomass together determine nutrient uptake per area unit, so a low nutrient concentration may offset a high biomass yield. Nonetheless, biomass yield affects nutrient uptake significantly more than nutrient concentration. Here, the cultivar HM-1 produced a more seed yield (Fig 2A) than Sapna and Garima. Also, the nutrient concentration in the seeds of HM-1 remained statistically at par, as presented in Table 1. Although numerically, it was less than in the seeds of Sapna and Garima, but not too less to counter the effect of seed yield on nutrient uptake in HM-1. In the case of nutrient uptake by stover, the cultivar Sapna, being statistically at par with Garima, recorded 14.7, 4.1, 11.2, 11.2, and 12.9% higher uptake of N, P, K, Zn, and Fe than HM-1 (Fig 3). This was directly linked with the significantly higher stover yield in cultivars Sapna and Garima over HM-1, as shown in Fig 2A. Whereas, the cultivar HM-1 was more efficient in total N and P uptake (seed + stover) than Sapna and Garima. However, the total K, Zn, and Fe uptake among lentil cultivars remained statistically similar. Total nutrient uptake is the sum of nutrient uptake by both seed and stover. Here, in this experiment, the contribution of nutrient uptake by seed in total uptake is more significant than that of stover. In this case, also, the higher total uptake of NP by cultivar HM-1 (Fig 4) was due to the higher uptake of NP in its seed than that of Sapna and Garima, as per Fig 3. Although their uptake in the stover of cultivars Sapna and Garima was greater, as shown in Fig 3. But the contribution of the stover in total uptake across the cultivars was only 21.6 to 28.0% for N and 37.3 to 45.8% for P. In contrast, P uptake by stover (Fig 3) has non-significant variations among the cultivars. As a result, the total N and P uptake were also higher in cultivar HM-1. A considerable variation in the uptake of macro as well as micronutrients among different lentil cultivars was also observed by Singh et al. [3], Gahoonia et al. [54], and Khatun et al. [55].

#### 4.1.3 Soil chemical properties

The data on soil pH, EC, OC, available N, P, and K, DPTA-extractable Zn, and Fe (Figs 5 and 6) showed that their values did not change significantly concerning initial values as well as from the effect of varietal selection. However, numerical variations were noted over the cultivars and growing years. Likewise, Karan et al. [56] observed that the available NPK and Zn in the soil exhibited a non-significant but variable trend in the soil ecosystem in the lentil cultivars. In another finding, Shylla et al. [57] reported that in the soil, the lentil cultivars IPL-406 and DPL 81 contained statistically similar SOC and available NP. Also, according to Nandan et al. [58], the chickpea cultivars showed negative variations in available PK content in the soil after crop harvest.

#### 4.1.4 Nutrient use efficiencies

The NUEs denote the crop yield improvement per unit of nutrient addition to the crop, thus driving productivity improvement gain by using nutrient input. Improving nitrogen uptake and reducing nutrient losses in soil, water, and the environment is critical to enhancing NUEs, productivity, economic efficiency, and environmental security [59]. In the present two-year experiment, the cultivar HM-1 has the most significant impact on PFP (24.27–24.59 kg kg^-1^), PNB (2.09–2.13 kg kg^-1^) and IUE (11.64–11.85 kg kg^-1^) of cumulative NPK in lentils compared to cultivars Sapna and Garima (Table 3). The ability of cultivar HM-1 to maintain a higher N inflow (N intake rate per unit root) than other cultivars may be the cause/mechanism of its superior nutritional efficiency. Thinner roots with higher surface areas in cultivar HM-1 explore the soil more extensively, perhaps increasing nutrient availability [60]. Genetic changes in central physiological and morphological features such as nutrition intake, metabolism, distribution, and remobilization account for varietal variability in nutrient utilization [61]. Individual morphological, anatomical, and biophysical traits include larger canopies with thicker leaves; larger leaf phloem transactional area, rapid solubilization and remobilization of nutrients from older to younger leaves, and lower dark respiration rates, which may be linked to lower NUEs of lentil cultivars Sapna and Garima. Nutrient transport within the plant system significantly impacts the amount of nutrients supplied to seeds and eventual economic production [62–64]. In this direction, two cultivars (Sapna and Garima) received a bulky amount of nutrients from the soil, but they couldn’t get them to seed and convert them to commercial yield, lowering the NUE.

### 4.2 Nutrient management

#### 4.2.1 Crop yield

Among diverse nutrient management practices, N10 produced significantly more seed (1.63–1.65 Mg ha^-1^), stover (1.96–2.02 Mg ha^-1^), and biological (3.59–3.62 Mg ha^-1^) yield than other practices (Fig 2A). However, seed, stover, and biological Yield in N10 were statistically similar to N4, N8, and N9. The main reason for increased crop production with N10 was an improvement in growth-attributing factors, followed by yield attributes such as pods per plant and seeds per pod. The improvement in growth and yield parameters and, consequently, crop yield in N10 resulted from the combined action of the major nutrients through the chemical and organic sources. They increased the availability of additional nutrients through their continuous slow-release and thus improved crop performance. The favourable growth conditions significantly impact yield attributes, resulting in a rise in crop output.

Furthermore, crop productivity is primarily determined by two factors: first, material precursors, and second, structural materials. Precursors are soil nutrients that plants exert through their roots, as well as primary photosynthates produced by green plant organs in the presence of light and CO_2_. Photosynthesis and plant nutrients combine to produce structural material that is a source and a sink of energy. Photosynthates are produced in chloroplasts containing chlorophyll, which contains nitrogen as one of its elements; hence, crop productivity rises with nitrogen levels. Earlier, Singh et al. [4], Niri et al. [65], and Singh et al. [66] also proved the significance of integrated use of organic and inorganic fertilizers towards improvement in the crop yield of lentils.

#### 4.2.2 Nutrient content, uptake and seed protein

The N concentration in the seed and stover of lentils was higher in N10, statistically equal with N9, N8, and N4 and significantly more than the remaining treatments (Tables 1 and 2). Also, the P content in seed and stover was higher in N10, but it has significant superiority only to the control plot. Adding nutrients to the soil through chemical fertilizers and VC significantly enhanced the uptake of different nutrients by seed and stover in the plot where no nutrients were applied. The maximum uptake of NPK by seed and stover was in N10 (Fig 3). This might be because the combined application of chemical fertilizers along with enough bulk of VC stimulates soil microbes and improves root growth by providing a congenial soil physical condition. This combined fertilization ensures the continuous supply of all essential nutrients directly to the crop or indirectly through checking the losses of nutrients from the soil solution, thereby increasing nutrient content, uptake and ultimately NUE. Vermicomposting increases the availability of P in soil solution for plant use. As a result, its content in the plant increased. The increase in P content in the plant (seed + stover) might also be due to the better buffering capacity of VC for incipient moisture stress and improved P availability to the plant. The results conformed to the findings of Arya et al. [67], Quddus et al. [68], and Sahu et al. [69]. The trend in seed protein content (Fig 2B) was similar to seed N content (Table 1). Because N is a primary constituent of protein, and with an increase in the rate of N application through organic and inorganic sources, seed protein synthesis increased due to improved N availability. The results confirm the findings of Subbian and Palaniappan [70]. The total uptake (seed + stover) of NPKZn and Fe was higher in N10 (Fig 4). As the total nutritional uptake is cumulative of both seed and stover uptake, their individual uptake directly affects the total uptake accordingly. Here also, the treatment N10 has more nutrient uptake in stover and seed (Fig 3); hence total uptake was also in favor of N10.

The foliar application of ZnSO_4_ and/or FeSO_4_ (0.5%) at pre-flowering and pod formation stages enriched the lentil seed and stover with Zn and Fe (Tables 1 and 2). They biofortified seeds with Zn by 7.0–7.7% and Fe by 9.9–10.5%, besides enrichment of stover by 6.3–7.7% (Zn) and 9.9–10.5% (Fe), over control. As foliar-applied Zn and Fe are phloem-mobile and highly water-soluble, they can thus be readily translocated into developing grains. It allows for their more significant and immediate uptake by plant leaves and reproductive plant parts. As a result, in Zn/Fe applied treatments, their concentration and uptake in the plants increase, as shown in Figs 3 and 4. The increased micronutrient concentration in seed and stover upon foliar biofortification with Zn and Fe at the reproductive stage has been reported by Nandan et al. [58] and Raghuwanshi et al. [71]. The uptake of Zn and Fe by seed and stover individually or together (Figs 3 and 4) was highest with treatment N10 owing to its higher biomass production (seed and stover yield) (Fig 2A) and the concentration of Zn and Fe (Tables 1 and 2). Because of this, their total uptake was also higher in N10. The results agree with the findings of Raghuwanshi et al. [71] and Ravi et al. [72].

#### 4.2.3 Soil chemical properties

Soil pH, EC, and soil organic carbon (SOC) were not found to be significantly changed by nutrient management practices in soil under lentil cultivation in this two-year study (Fig 5). Although numerically, their values changed as compared to the control plot. Numerically, the soil pH and EC were slightly lower than their initial values in plots supplied with VC owing to the release of organic acids upon its decomposition. While their values somewhat increased with chemical fertilization over control due to the higher soluble salts in synthetic fertilizers. Likewise, the soil incorporation of VC slightly improved the soil C stock and its extent varied with the amount of VC used. The findings of Ansari et al. [73] and Shukla et al. [74] confirm and support the outcome.

The stock of available soil N (Fig 6) improved after crop harvest in N10 (149.3 kg ha^-1^) during the first year compared to the initial value before the start of the experiment (137.0 kg ha^-1^). This might be due to N left behind in the soil after crop harvest owing to applying chemical fertilizers coupled with organic manure and unquantified and uncalculated BNF by the lentil crop itself [75]. Afterwards, N concentration decreased again during the fallow period of the hot summer due to nutritional losses upon decreased SOC and increased erosion as structural stability declined. Available N significantly increased in the soil after harvest (Fig 6). The maximum improvement in N status was found from the combined application of VC with a reduced dose of chemical N and the total amount of chemical P (N10, N9, N8, and N4). It may be due to the mineralization of VC in soil and the initial availability of N through synthetic fertilizers. The vermicomposting promoted by earthworms is a biological storehouse of all the essential plant nutrients required for crop growth and development. Thus, it is a firm soil and plant health promoter. Vermicomposting upgrades soil’s physicochemical and biological environment, including soil C stock, soil structure, porosity, and water holding capacity, besides reducing soil compaction and crusting. The availability of most essential plant nutrients increased owing to their slight reduction in soil pH towards neutralization and improvement in the soil’s cation exchange capacity. These observations are in close agreement with the findings of Singh et al. [76], who indicated that the judicious integration of organic and inorganic sources of nutrition significantly improved the available soil N.

In contrast, none of the nutrient management practices significantly affected available PKZn and Fe in the soil in two years. However, adding vermicompost slightly improved their values. It might be due to the greater solubilization and mobilization of fixed native soil P, vigorous root proliferation, and contribution through biomass. The solubilizing action of various organic acids released during the decomposition of VC has a better capacity to hold K in the soluble form. Various studies have noted the improved availability of nutrients in soil upon vermicomposting due to its mineralization and subsequent release of nutrients in soil solution [77–80]. The results of the study carried out by Nandan et al. [58] indicated that the soil parameters after legume crops’ harvest showed non-significant variation with PKZn and Fe except for available N in the soil, which was significantly influenced by the combined application of inorganic fertilizers and organic manure. The results also confirmed the findings of Sahai et al. [81] and Karmakar et al. [82].

#### 4.2.4 Nutrient use efficiencies

Efficient nutrient management is the key to exploring lentil crops’ potential for improving NUEs. Applying more nutrients does not necessarily mean that the NUEs are improved. In general, NUEs decrease as the application rate of chemical fertilizers increases. That’s why it is crucial to optimize chemical fertilizers, but they should be sustainably integrated with organic sources to avoid yield reduction. In the same line, we recorded the reduced NUEs for NPK with an increase in the rate of NPK. The impact of N10 on improving AE (7.26–7.97 kg kg^-1^) and ARE (87–93%) of lentils was significantly higher. Whereas, in the case of PE, the impact of N4 (9.59, 14.52%) was the greatest over the remaining treatments (Table 3).

Similarly, the N7 has the highest PFP (23.47–23.35 kg kg^-1^) and PNB (2.26–2.29 kg kg^-1^). The increased AE and ARE in N10 for NPK could be due to the yield increment per unit of nutrient applied. The higher yield was linked to the beneficial effect of combining organic manures and inorganic fertilizers on soil health, including a higher nutritional level, which influenced the growth and yield-attributing characteristics of the crop. Furthermore, proper vermicompost decomposition provided plant nutrients directly to plants, resulting in a more favourable soil environment, improved nutrient balance, indigenous soil nutrient supply, and improved crop growth and yield [83]. The improved AE and ARE of lentils were directly attributed to greater seed yields. Fageria and Baligar [84] also reported that substantial NUEs could be achieved if the yield increment per unit nutrient supplied is high due to reduced losses and enhanced nutrient uptake. The findings support those of Mondal et al. [53]; Kumar et al. [85]; Dass et al. [86], and Haile et al. [87]. Furthermore, the enhanced PE for NPK could be linked to plants’ ability to efficiently convert acquired NPK in source to the production of economic Yield under N4. This helps to determine the source and amount of mineral nutrition that must be consumed, mobilized, used, and converted into profitable biomass. Similarly, the increase in IUE for NPK in N2 (11.59–11.64 kg^-1^) was linked to developing a significant quantity of seed yield per unit of NPK uptake.

## 5. Conclusions

This experiment explores the potential of lentil cultivars for continual and improved nutrient delivery from organic sources coupled with synthetic fertilizers to improve crop yield, NUEs, soil, environment, and human health protection. Based on this, it is concluded that the lentil cultivar HM-1 recorded significantly higher seed yield (1.59–1.61 Mg ha^-1^), uptake of NPKZn and Fe in seed, and NUEs for NPK compared to cultivars Sapna and Garima. On the other hand, seed yield (1.62–1.65 Mg ha^-1^), seed protein as well as the concentration and uptake of macro and micronutrients in seed and stover were highest with an application of 50% RDN + 100% RDP + VC at 1 t ha^-1^ + 0.5% ZnSO_4_ + 0.5% FeSO_4_ foliar spray (N10). Available N in soil increased from its initial value of 137.0 kg ha^-1^ to 145.3 kg ha^-1^ in N10 and decreased to 124.3 kg ha^-1^ in control after two years of experimentation. N10 has a more significant impact on AE for NPK (7.26–7.97 kg kg^-1^). Hence, the lentil cultivar HM-1 can be successfully grown in the semi-arid region of northern India by substituting 50% RDN with organic manures, *i*.*e*., vermicompost, without compromising crop productivity and soil fertility. The interest of farmers must be turned toward a simple technique of INM, which is an acceptable option. Farmers may readily adopt this environmentally friendly practice, which is cost-effective, and produce crops with improved yields and quality traits while making a reasonable profit.

## Supporting information

S1 File (DOCX)
