# Supplementary material for: Efficient nutrient management for enhancing crop productivity, quality and nutrient dynamics in lentil (Lens culinaris Medik.) in the semi-arid region of northern India
Source: PLoS One. 2023 Feb 10;18(2):e0280636. doi: 10.1371/journal.pone.0280636 (PMC9916625; doi:10.1371/journal.pone.0280636)
Supplement: S1 File — (DOCX) [file pone.0280636.s001.docx]

**Raw data used to generate Figures/Graphs etc.**

**Dear Editor,**

We are attaching herewith the values/data used for the preparation of figures/graphs in the manuscript. Data includes Fig. 2 to Fig. 6. In Fig. 7 and 8, correlation and PCA analysis was carried out using the data of Tables 5 and 6 (attached herewith). Apart from this, no other raw data were used in the manuscript.

**Table 1: Crop yield and seed protein content of lentil cultivars under different nutrient management levels**

| **Treatment** | **Seed yield**  **(Mg ha^-1^)** | | **Stover yield**  **(Mg ha^-1^)** | | **Biological yield**  **(Mg ha^-1^)** | | **Seed protein content (%)** | |
| --- | --- | --- | --- | --- | --- | --- | --- | --- |
|  | **2016** | **2017** | **2016** | **2017** | **2016** | **2017** | **2016** | **2017** |
| **Cultivars** | | | | | | | | |
| Sapna | 1.33 | 1.31 | 1.86 | 1.90 | 3.19 | 3.21 | 27.0 | 27.0 |
| Garima | 1.30 | 1.30 | 1.82 | 1.87 | 3.12 | 3.17 | 26.5 | 26.6 |
| HM-1 | 1.59 | 1.61 | 1.68 | 1.73 | 3.27 | 3.34 | 26.1 | 26.3 |
| SEm± | 0.02 | 0.02 | 0.03 | 0.03 | 0.03 | 0.05 | 0.36 | 0.41 |
| LSD (­*p≤*0.05) | 0.09 | 0.09 | 0.13 | 0.13 | NS | NS | NS | NS |
| **Nutrient management practices** | | | | | | | | |
| N_1_ | 1.06 | 1.07 | 1.42 | 1.43 | 2.48 | 2.50 | 22.0 | 22.1 |
| N_2_ | 1.32 | 1.35 | 1.75 | 1.80 | 3.07 | 3.15 | 25.9 | 26.2 |
| N_3_ | 1.19 | 1.21 | 1.59 | 1.62 | 2.78 | 2.83 | 24.0 | 24.1 |
| N_4_ | 1.54 | 1.56 | 1.94 | 2.00 | 3.48 | 3.56 | 28.4 | 28.6 |
| N_5_ | 1.38 | 1.38 | 1.77 | 1.81 | 3.15 | 3.19 | 26.3 | 26.4 |
| N_6_ | 1.35 | 1.36 | 1.76 | 1.81 | 3.11 | 3.17 | 26.1 | 26.3 |
| N_7_ | 1.41 | 1.40 | 1.77 | 1.82 | 3.18 | 3.22 | 26.4 | 26.5 |
| N_8_ | 1.60 | 1.62 | 1.95 | 2.01 | 3.55 | 3.63 | 28.7 | 28.8 |
| N_9_ | 1.57 | 1.60 | 1.95 | 2.01 | 3.52 | 3.61 | 28.6 | 28.6 |
| N_10_ | 1.63 | 1.65 | 1.96 | 2.02 | 3.59 | 3.67 | 28.8 | 28.9 |
| SEm± | 0.04 | 0.05 | 0.06 | 0.06 | 0.07 | 0.07 | 0.67 | 0.69 |
| LSD (­*p≤*0.05) | 0.12 | 0.14 | 0.16 | 0.17 | 0.21 | 0.20 | 1.91 | 1.95 |

**Table 2: Nutrient uptake by seed of lentil as influenced by cultivar selection and nutrient management levels**

| **Treatment** | **Nitrogen (kg ha^-1^)** | | **Phosphorus (kg ha^-1^)** | | **Potassium (kg ha^-1^)** | | **Zinc (g ha^-1^)** | | **Iron (g ha^-1^)** | |
| --- | --- | --- | --- | --- | --- | --- | --- | --- | --- | --- |
|  | **2016** | **2017** | **2016** | **2017** | **2016** | **2017** | **2016** | **2017** | **2016** | **2017** |
| **Cultivar** | | | | | | | | | | |
| Sapna | 57.7 | 57.8 | 5.6 | 5.7 | 11.4 | 11.7 | 50.6 | 50.7 | 137.5 | 138.3 |
| Garima | 55.9 | 55.9 | 5.2 | 5.3 | 11.0 | 11.6 | 49.8 | 49.9 | 135.4 | 136.1 |
| HM 1 | 67.2 | 67.6 | 6.8 | 7.0 | 13.8 | 13.9 | 60.4 | 61.1 | 162.5 | 165.1 |
| SEm± | 1.4 | 1.7 | 0.11 | 0.12 | 0.28 | 0.33 | 1.1 | 1.2 | 4.0 | 3.8 |
| CD at 5% | 5.5 | 6.6 | 0.41 | 0.46 | 1.12 | 1.28 | 4.5 | 4.9 | 15.8 | 15.0 |
| **Nutrient management** | | | | | | | | | | |
| N1 | 37.4 | 36.2 | 3.82 | 3.79 | 8.96 | 8.81 | 39.0 | 37.9 | 104.5 | 101.8 |
| N2 | 50.7 | 50.9 | 5.42 | 5.53 | 11.23 | 11.51 | 49.1 | 48.8 | 130.81 | 132.7 |
| N3 | 49.9 | 48.9 | 4.65 | 4.74 | 10.52 | 10.45 | 44.5 | 43.9 | 117.7 | 117.5 |
| N4 | 67.6 | 68.5 | 6.79 | 6.83 | 13.44 | 13.72 | 57.5 | 57.9 | 153.4 | 155.8 |
| N5 | 58.3 | 58.3 | 5.66 | 6.02 | 11.80 | 12.04 | 54.6 | 54.8 | 136.9 | 136.8 |
| N6 | 57.6 | 57.7 | 5.56 | 5.66 | 11.49 | 11.86 | 49.9 | 50.2 | 147.1 | 149.3 |
| N7 | 63.7 | 64.2 | 5.91 | 5.89 | 12.03 | 12.15 | 55.8 | 55.6 | 153.2 | 153.2 |
| N8 | 73.1 | 74.3 | 6.89 | 7.09 | 13.99 | 14.30 | 63.4 | 64.4 | 158.7 | 161.8 |
| N9 | 71.9 | 73.1 | 6.75 | 7.03 | 13.61 | 14.13 | 58.3 | 59.3 | 171.1 | 175.2 |
| N10 | 74.9 | 75.7 | 7.17 | 7.55 | 14.06 | 14.56 | 64.6 | 65.5 | 177.9 | 181.2 |
| SEm± | 3.0 | 2.6 | 0.25 | 0.29 | 0.51 | 0.59 | 1.7 | 2.1 | 6.4 | 7.8 |
| LSD (­*p≤*0.05) | 8.6 | 7.5 | 0.72 | 0.81 | 1.45 | 1.66 | 4.8 | 6.0 | 18.3 | 22.2 |

**Table 3: Nutrient uptake by of lentil as influenced by cultivar selection and nutrient management levels**

| **Treatment** | **Nitrogen (kg ha^-1^)** | | **Phosphorus (kg ha^-1^)** | | **Potassium (kg ha^-1^)** | | **Zinc (g ha^-1^)** | | **Iron (g ha^-1^)** | |
| --- | --- | --- | --- | --- | --- | --- | --- | --- | --- | --- |
|  | **2016** | **2017** | **2016** | **2017** | **2016** | **2017** | **2016** | **2017** | **2016** | **2017** |
| **Cultivar** | | | | | | | | | | |
| Sapna | 21.32 | 22.19 | 4.24 | 4.50 | 27.10 | 29.21 | 46.9 | 49.7 | 402.7 | 418.2 |
| Garima | 20.49 | 21.41 | 4.32 | 4.62 | 26.98 | 28.13 | 45.7 | 48.8 | 390.9 | 409.9 |
| HM 1 | 18.53 | 19.40 | 4.04 | 4.36 | 25.12 | 25.51 | 42.1 | 44.8 | 355.7 | 371.6 |
| SEm± | 0.52 | 0.44 | 0.13 | 0.15 | 0.47 | 0.57 | 1.7 | 1.3 | 8.3 | 9.5 |
| CD at 5% | 1.72 | 1.77 | NS | NS | 1.76 | 1.96 | 3.5 | 3.7 | 30.5 | 32.8 |
| **Nutrient management** | | | | | | | | | | |
| N1 | 12.40 | 13.98 | 2.83 | 3.00 | 20.64 | 21.04 | 34.6 | 36.0 | 293.8 | 298.2 |
| N2 | 19.14 | 19.72 | 4.03 | 4.33 | 25.46 | 26.88 | 42.9 | 45.5 | 362.8 | 376.0 |
| N3 | 15.70 | 17.54 | 3.49 | 3.71 | 24.17 | 24.77 | 39.2 | 41.3 | 329.8 | 340.0 |
| N4 | 23.45 | 24.47 | 4.82 | 5.01 | 28.82 | 30.35 | 47.6 | 50.9 | 378.2 | 415.7 |
| N5 | 19.52 | 20.01 | 4.07 | 4.52 | 25.89 | 27.13 | 45.9 | 48.8 | 367.3 | 377.3 |
| N6 | 19.35 | 19.70 | 4.04 | 4.32 | 25.65 | 27.15 | 43.1 | 45.7 | 401.8 | 418.9 |
| N7 | 19.75 | 19.96 | 4.28 | 4.37 | 26.00 | 27.05 | 46.1 | 49.1 | 403.7 | 419.6 |
| N8 | 23.95 | 24.80 | 4.66 | 5.23 | 28.99 | 30.56 | 50.8 | 54.4 | 403.6 | 420.0 |
| N9 | 23.73 | 24.72 | 4.87 | 5.20 | 28.90 | 30.50 | 47.8 | 51.1 | 443.6 | 466.0 |
| N10 | 24.14 | 25.09 | 4.90 | 5.24 | 29.08 | 30.71 | 51.1 | 54.8 | 446.3 | 466.8 |
| SEm± | 0.84 | 0.88 | 0.18 | 0.23 | 1.15 | 1.35 | 1.6 | 1.9 | 17.0 | 21.3 |
| LSD (­*p≤*0.05) | 2.39 | 2.43 | 0.41 | 0.58 | 2.15 | 2.26 | 4.5 | 4.7 | 48.3 | 50.1 |

**Table 4: Total nutrient uptake (seed + stover) of lentil as influenced by cultivar selection and nutrient management levels**

| **Treatment** | **Nitrogen (kg ha^-1^)** | | **Phosphorus (kg ha^-1^)** | | **Potassium (kg ha^-1^)** | | **Zinc (g ha^-1^)** | | **Iron (g ha^-1^)** | |
| --- | --- | --- | --- | --- | --- | --- | --- | --- | --- | --- |
|  | **2016** | **2017** | **2016** | **2017** | **2016** | **2017** | **2016** | **2017** | **2016** | **2017** |
| **Cultivar** | | | | | | | | | | |
| Sapna | 79.0 | 79.2 | 9.47 | 9.83 | 38.14 | 40.86 | 85.2 | 99.6 | 538 | 554 |
| Garima | 76.4 | 77.3 | 9.90 | 10.29 | 38.26 | 39.73 | 83.9 | 99.4 | 528 | 548 |
| HM 1 | 85.7 | 87.9 | 10.82 | 11.39 | 39.02 | 39.31 | 80.1 | 105.9 | 518 | 536 |
| SEm± | 1.5 | 2.0 | 0.19 | 0.20 | 0.59 | 1.02 | 1.4 | 2.4 | 10 | 11 |
| CD at 5% | 5.9 | 7.8 | 0.74 | 0.77 | NS | NS | NS | NS | NS | NS |
| **Nutrient management** | | | | | | | | | | |
| N1 | 49.8 | 50.1 | 6.65 | 6.78 | 29.60 | 29.86 | 71.5 | 73.9 | 398 | 400 |
| N2 | 69.8 | 70.5 | 8.91 | 9.24 | 35.39 | 36.28 | 80.0 | 94.3 | 493 | 508 |
| N3 | 65.2 | 66.5 | 8.68 | 9.06 | 35.98 | 37.34 | 76.6 | 85.2 | 447 | 457 |
| N4 | 88.1 | 90.0 | 10.83 | 11.15 | 39.09 | 40.87 | 84.9 | 108.9 | 531 | 574 |
| N5 | 77.8 | 78.2 | 9.73 | 10.54 | 37.69 | 39.16 | 85.4 | 103.6 | 504 | 514 |
| N6 | 77.1 | 77.2 | 9.84 | 10.03 | 37.48 | 38.91 | 80.3 | 96.0 | 548 | 565 |
| N7 | 84.0 | 83.6 | 10.74 | 11.09 | 40.93 | 42.50 | 85.6 | 104.7 | 556 | 572 |
| N8 | 97.0 | 99.1 | 11.76 | 12.10 | 42.80 | 44.86 | 90.5 | 118.8 | 562 | 581 |
| N9 | 95.7 | 97.8 | 11.41 | 12.26 | 42.69 | 44.63 | 85.1 | 110.4 | 614 | 641 |
| N10 | 99.0 | 100.8 | 12.07 | 12.79 | 43.05 | 45.26 | 90.8 | 120.3 | 624 | 647 |
| SEm± | 3.2 | 2.5 | 0.38 | 0.40 | 1.56 | 1.41 | 2.1 | 2.9 | 21 | 26 |
| LSD (­*p≤*0.05) | 9.0 | 7.3 | 1.08 | 1.12 | 4.41 | 4.01 | 5.9 | 8.2 | 60 | 73 |

**Table 5: Soil pH, EC and organic carbon as influenced by cultivar selection and nutrient management levels**

| **Treatment** | **pH (1:2)** | | | **EC (dS m^-1^)** | | | **Organic carbon (%)** | | |
| --- | --- | --- | --- | --- | --- | --- | --- | --- | --- |
|  | **2016**  **At harvest** | **2017**  **Initial** | **2017**  **At harvest** | **2016**  **At harvest** | **2017**  **Initial** | **2017**  **At harvest** | **2016**  **At harvest** | **2017**  **Initial** | **2017**  **At harvest** |
| **Cultivar** | | | | | | | | |  |
| Sapna | 8.18 | 8.16 | 8.14 | 0.50 | 0.50 | 0.52 | 0.42 | 0.41 | 0.42 |
| Garima | 8.20 | 8.15 | 8.15 | 0.52 | 0.51 | 0.49 | 0.42 | 0.41 | 0.41 |
| HM 1 | 8.17 | 8.11 | 8.15 | 0.50 | 0.48 | 0.53 | 0.41 | 0.41 | 0.41 |
| SEm± | 0.04 | 0.06 | 0.07 | 0.01 | 0.01 | 0.01 | 0.01 | 0.005 | 0.003 |
| CD at 5% | NS | NS | NS | NS | NS | NS | NS | NS | NS |
| **Nutrient management** | | | | | | | | | |
| N1 | 8.21 | 8.20 | 8.18 | 0.51 | 0.50 | 0.50 | 0.40 | 0.39 | 0.38 |
| N2 | 8.30 | 8.24 | 8.31 | 0.53 | 0.53 | 0.55 | 0.41 | 0.40 | 0.40 |
| N3 | 8.05 | 8.02 | 7.98 | 0.48 | 0.47 | 0.46 | 0.44 | 0.42 | 0.45 |
| N4 | 8.11 | 8.04 | 8.04 | 0.50 | 0.52 | 0.55 | 0.43 | 0.42 | 0.43 |
| N5 | 8.29 | 8.29 | 8.32 | 0.54 | 0.51 | 0.56 | 0.42 | 0.41 | 0.42 |
| N6 | 8.26 | 8.25 | 8.29 | 0.55 | 0.53 | 0.54 | 0.41 | 0.40 | 0.41 |
| N7 | 8.25 | 8.22 | 8.28 | 0.52 | 0.50 | 0.53 | 0.41 | 0.40 | 0.40 |
| N8 | 8.17 | 8.10 | 8.05 | 0.51 | 0.50 | 0.49 | 0.42 | 0.41 | 0.43 |
| N9 | 8.08 | 8.03 | 8.01 | 0.49 | 0.48 | 0.49 | 0.43 | 0.42 | 0.44 |
| N10 | 8.10 | 8.06 | 7.99 | 0.48 | 0.48 | 0.50 | 0.43 | 0.42 | 0.44 |
| SEm± | 0.07 | 0.16 | 0.17 | 0.02 | 0.02 | 0.03 | 0.01 | 0.01 | 0.01 |
| LSD (­*p≤*0.05) | NS | NS | NS | NS | NS | NS | NS | NS | NS |

**Table 6: Status of soil available nutrients as influenced by cultivar selection and nutrient management levels**

| **Treatment** | **N (kg ha^-1^)** | | | **P_2_O_5_ (kg ha^-1^)** | | | **K_2_O (kg ha^-1^)** | | |
| --- | --- | --- | --- | --- | --- | --- | --- | --- | --- |
|  | **2016**  **At harvest** | **2017 Initial** | **2017**  **At harvest** | **2016**  **At harvest** | **2017**  **Initial** | **2017**  **At harvest** | **2016**  **At harvest** | **2017**  **Initial** | **2017**  **At harvest** |
| **Cultivar** | | | | | | | | | |
| Sapna | 145.7 | 138.0 | 141.7 | 14.98 | 13.92 | 14.06 | 406.8 | 402.8 | 397.3 |
| Garima | 144.1 | 137.6 | 141.0 | 15.16 | 14.57 | 14.49 | 401.8 | 403.8 | 399.1 |
| HM 1 | 143.9 | 136.3 | 139.7 | 15.55 | 14.23 | 14.76 | 397.6 | 396.1 | 395.3 |
| SEm± | 2.3 | 1.6 | 3.0 | 0.17 | 0.21 | 0.19 | 5.9 | 5.7 | 5.8 |
| CD at 5% | NS | NS | NS | NS | NS | NS | NS | NS | NS |
| **Nutrient management** | | | | | | | | | |
| N1 | 132.6 | 127.8 | 124.3 | 13.56 | 13.41 | 12.78 | 397.4 | 396.2 | 392.5 |
| N2 | 144.2 | 137.2 | 141.3 | 15.02 | 14.21 | 14.52 | 398.4 | 397.2 | 395.5 |
| N3 | 139.2 | 134.2 | 136.4 | 14.74 | 14.16 | 14.35 | 407.7 | 406.6 | 402.8 |
| N4 | 149.1 | 140.3 | 145.3 | 15.30 | 14.29 | 14.75 | 404.7 | 403.5 | 400.1 |
| N5 | 144.3 | 137.3 | 141.5 | 15.05 | 14.23 | 14.54 | 403.0 | 401.8 | 395.6 |
| N6 | 144.3 | 137.2 | 141.4 | 15.05 | 14.22 | 14.58 | 400.0 | 398.8 | 392.7 |
| N7 | 144.4 | 137.3 | 141.5 | 15.06 | 14.23 | 14.60 | 397.0 | 395.8 | 395.0 |
| N8 | 149.2 | 140.4 | 145.4 | 15.32 | 14.30 | 14.69 | 403.2 | 402.0 | 398.3 |
| N9 | 149.1 | 140.4 | 145.3 | 15.32 | 14.29 | 14.70 | 404.3 | 403.1 | 399.2 |
| N10 | 149.3 | 140.5 | 145.5 | 15.32 | 14.30 | 14.73 | 405.3 | 404.2 | 400.3 |
| SEm± | 3.9 | 3.7 | 4.0 | 0.54 | 0.51 | 0.47 | 11.1 | 12.7 | 13.5 |
| LSD (­*p≤*0.05) | 11..0 | 10.7 | 11.4 | NS | NS | NS | NS | NS | NS |
